# *N*-Lipidated Amino Acids and Peptides Immobilized on Cellulose Able to Split Amide Bonds

**DOI:** 10.3390/ma12040578

**Published:** 2019-02-14

**Authors:** Justyna Fraczyk, Zbigniew J. Kaminski

**Affiliations:** Institute of Organic Chemistry, Lodz University of Technology, Zeromskiego 116, 90-924 Lodz, Poland; zbigniew.kaminski@p.lodz.pl

**Keywords:** synzyme, catalytic activity, peptides immobilized on the solid support, catalytic pocket, molecular mimicry, triazine coupling reagent

## Abstract

*N*-lipidated short peptides and amino acids immobilized on the cellulose were used as catalysts cleaved amide bonds under biomimetic conditions. In order to select catalytically most active derivatives a library of 156 *N*-lipidated amino acids, dipeptides and tripeptides immobilized on cellulose was obtained. The library was synthesized from serine, histidine and glutamic acid peptides *N*-acylated with heptanoic, octanoic, hexadecanoic and (E)-octadec-9-enoic acids. Catalytic efficiency was monitored by spectrophotometric determination of *p*-nitroaniline formed by the hydrolysis of a 0.1 M solution of Z-Leu-NP. The most active 8 structures contained tripeptide fragment with 1-3 serine residues. It has been found that incorporation of metal ions into catalytic pockets increase the activity of the synzymes. The structures of the 17 most active catalysts selected from the library of complexes obtained with Cu^2+^ ion varied from 16 derivatives complexed with Zn^2+^ ion. For all of them, a very high reaction rate during the preliminary phase of measurements was followed by a substantial slowdown after 1 h. The catalytic activity gradually diminished after subsequent re-use. HPLC analysis of amide bond splitting confirmed that substrate consumption proceeded in two stages. In the preliminary stage 24–40% of the substrate was rapidly hydrolysed followed by the substantially lower reaction rate. Nevertheless, using the most competent synzymes product of hydrolysis was formed with a yield of 60–83% after 48h under mild and strictly biomimetic conditions.

## 1. Introduction

Macromolecular catalysis is a rapidly developing field. Some of the most fascinating catalysts are synthetic enzymes, known as synzymes, which mimic the activity, function and properties of natural enzymes. Representative members include chemzymes [1], artificial enzymes [2], artificial metalloenzymes [3], abzymes, nanozymes [4,5], artificial ribozymes, aptazymes [6] and transition-state analogues imprinted in polymers [7]. At the same time, synzymes are able to overcome many of the limitations of natural enzymes, such as low operational stability, limited selectivity, high costs associated with preparation and purification, incompatibility with solvents, as well as inability to function under abiotic conditions or in a wide range of non-natural reactions. Even in the case of synzymes obtained in the least assaulting processes, based on enzyme immobilization, the catalytic function, which transforms the starting substrates into products within the desired time and space, can be modulated for preferred activity, selectivity and substrate specificity.

The other, non-catalytic functions of this group of synzymes are to facilitate the separation of the catalyst from the products, to control the process as well as to ensure optimal conditions for the possible reuse of the catalyst. Non-catalytic functions are primarily related to the geometric properties of the immobilization carrier, such as shape, size, length and thickness [8]. Notwithstanding the many advantages of enzymes immobilized on solid supports, the carrier itself causes so-called “dilution of activity.” The carrier usually accounts for between 90% and 99.9% by weight of the immobilized enzyme. Its presence increases the reaction time and lowers the efficiency of the reaction [9]. Moreover, immobilization often leads to a drop in activity (of up to 50%) relative to the native enzyme, which is particularly noticeable in systems with high loading on a solid carrier [10]. 

Other synzymes were designed de novo and their active structure was composed from small fragments, to obtain a functional group already identified in the active centres of enzymes. The fragments were subsequently arranged by attaching them to the macromolecular carrier [11,12]. 

Many natural proteases, such as serine proteases, aspartic proteases and cysteine proteases, hydrolyse proteins using only those functional groups which occur in amino-acid side chains. Stimulated attempts to design and prepare organic catalyst systems using functional groups typical for amino acids have been made [13,14,15,16]. The first “artificial” protease contained three salicylate residues as the active centre [17]. Hydrolysis of the peptide bond was associated with the presence of carboxylic residues which mimicked aspartic protease [18]. Another artificial protease was obtained by introducing two or more imidazole residues, forming an active site, into the cross-linked polystyrene [19] or polyacrylamide [20]. In both cases, the artificial proteases used proteinogenic amino acid functional groups in their active centres. It is also possible to design an active artificial protease centre that contains functional groups characteristic of unnatural amino acids, which are not found in proteinogenic amino acids [21]. 

Even very short peptides, containing amino acids typical for the active centres of enzyme, can self-assemble into structures with a variety of physical and structural properties, promoting the creation of complex systems with emergent catalytic properties. Some short peptides have shown high catalytic activity in model reactions, reaching efficiencies comparable to those of natural enzymes [22,23,24,25]. Supramolecular peptidic tertiary structures containing specific functional groups of amino acid side-chains were formed in noncovalent interactions, ensuring catalytic activity and the ability to bind cofactors such as metal ions [26]. The disadvantage of self-assembled peptidic structures formed by networks of weaker noncovalent bonds is their tendency to decompose under relatively mild conditions. 

The stability of catalytic systems can be increased by depositing the catalyst on or inside a solid carrier. Co-assembled derivatives of the catalytic triad Fmoc-FFH, Fmoc-FFS and Fmoc-FFD (Fmoc- 9-fluorenylmethyloxycarbonyl) formed peptide-based nanofibers, providing a supramolecular framework which exposed the imidazol ring as well as hydroxy and carboxylic groups. Such systems have been shown to be able to effectively catalyse amide bond hydrolysis [27]. 

It has also been shown that *N*-lipidated peptides supported on a cellulose matrix are able to create cavities that mimic interactions inside the bonding pockets of receptor/enzyme and natural ligands/substrates [28,29]. The structure of the cellulose solid matrix determines the regularity of the distribution of functional groups on the surface of the biopolymer and the distance between them. Cellulose microfibrils oriented along the longitudinal axis of the fibrils are held together by interchain and intrachain hydrogen bonds [30,31,32] forming crystalline [33,34] and amorphous regions [35,36,37]. Immobilized on cellulose peptides form cavities as a result of the self-assembly process, in which weak bonds are binding ligand molecules [38] and conformational changes [39] are induced in the peptide backbone and side chains of the amino-acid residues. The interactions between the binding cavity and the ligand (a substrate in the case of the molecular enzyme) are highly selective. The conformational freedom of the peptide fragment and the diversity of the functional groups in the side chains of the amino acid residues ensure flexibility of the binding cavity. It is also providing the possibility of optimized adaptation to the requirements of the bound ligand/substrate. This features of the *N*-lipidated peptides on the one hand are advantages and on the other hand disadvantages. The conformational freedom of short peptides without an innate ability to form ordered spatial structures causes that only under highly favourable conditions is it possible to form stable supramolecules composed from the molecular receptor and ligand, which makes it necessary to search the libraries of *N*-lipidated peptides to find the optimal structure of molecular receptor for a given ligand. On the other hand, the structural diversity of libraries of *N*-lipidated peptides makes it possible to find structures that form stable molecular receptors with diverse ligands. At this point, the lack of stable spatial structures of *N*-lipidated peptides causes that, as a result of self-assembly under favourable conditions, they are able to change the conformation induced by the ligand [38,39]. The results of preliminary studies [40] confirmed that *N*-lipidiated peptides immobilized on cellulose were capable of catalysing the hydrolysis and transesterification of *p*-nitrophenyl esters of *N*-protected peptides [41]. This means that the *N*-lipidated peptides immobilized on cellulose in a first step were able to form stable molecular complexes with *p*-nitrophenyl esters, which were subsequently cleaved due to the presence in the peptide fragment of the amino acid residues present in the catalytic triads of the proteases and finally to expel the reaction products to attend the next catalytic cycle. The results of these studies raised another question as to whether such structures would be able to hydrolyse the amide (peptide) bond. While the hydrolysis of the ester bond occurs relatively simply, hydrolysis of the amide bond under mild conditions (room temperature, near-neutral pH) is in principle highly improbable without appropriate catalyst. This prompted us to investigate: (i) the possibility of using *N*-lipidated amino acids, di- and tripeptides containing serine, histidine and glutamic acid residues as catalysts for peptide bond hydrolysis; (ii) the effect of a fatty acid residue on the efficiency of hydrolysis by synzymes containing amino acid residues with an active centre of serine proteases and (iii) whether it is possible to modulate the efficiency of the catalytic process using divalent metal ions acting as cofactors for natural enzymes. 

## 2. Materials and Methods

The procedures described below are analogous to those used for the synthesis of molecular receptors and chemzymes with esterase activity [41]. Whatman 7 (Sigma-Aldrich) (cellulose was used as a solid support for the synthesis of an array of molecular synzymes. Fmoc-protected amino acids were purchased form Novabiochem. Thin-layer chromatography experiments were performed on silica gel (Merck; 60 A F254). Spots were visualized with UV light (254 and 366 nm) and with 1% ethanolic 4-(4-nitrobenzyl)pyridine (NBP) (Sigma-Aldrich).

Melting points were determined using a Buchi apparatus, model 510 (BÜCHI Labortechnik GmbH, Essen, Germany).

Analytical RP-HPLC was performed with a Waters 600S HPLC system (Waters 2489 UV/VIS detector, Waters 616 pump, Waters 717 plus autosampler (Waters Poland, Warsaw, Poland) HPLC manager software from Chromax) (POL-LAB, Warsaw, Poland) using a C18 column (25 cm 9 4.6 mm, 5 mm; Sigma). HPLC was performed with a gradient of 0.1% trifluoroacetic acid (TFA) (Sigma-Aldrich) in H_2_O (A) and 0.08% TFA in acetonitrile (MeCN) (Baker) (B), at a flow rate of 1 mL/min with UV detection at 254 nm, t_R_ in min.

^1^H- and ^13^C-NMR spectra were recorded with a Bruker Avance DPX 250 (250 MHz) (Bruker Polska, Poznan, Poland) spectrometer, with chemical shifts [ppm] relative to TMS used as an internal standard. Multiplicities are marked as s = singlet, d = doublet, t = triplet, q = quartet, quint. = quintet, m = multiplet.

### 2.1. Functionalization of Whatman 7 Cellulose Sheets Using 2,4-Dichloro-6-Methoxy-1,3,5-Triazine (DCMT)

Whatman-7 filter paper (3 × 5 cm) was pre-functionalized by submerging 160 sheets in 1M NaOH aq. solution (400 mL) with gentle shaking (50 rpm) for 15 min (PSU-20i, Multi–functional Orbital Shaker, Biosan, Riga, Latvia). The excess solvent was removed, then the wet cellulose sheets were submerged in a suspension of finely ground sodium bicarbonate, 1M solution of DCMT (68 g) in tetrahydrofuran (THF) (400 mL) (Avantor), with gentle shaking for 45 min at room temperature. The sheets were then washed with 2 × THF (300 mL) for 10 min, 2 × acetone (300 mL), 2 × acetone:H_2_O 1:1 (400 mL), 2 × acetone (400 mL) (Avantor) and 1 × dichloromethane (DCM) (300 mL) (Avantor). The remaining solvent was removed and the functionalized filter papers were dried in a vacuum desiccator over P_2_O_5_ and KOH to constant mass.

Elemental analysis (VARIO EL III, Elementar, Langenselbold, Germany):

1. for starting cellulose: %N 0.00–0.05%; Cl 0.01–0.05.
2. for cellulose with attached chloro-dimethoxy-1,3,5-triazine: %N 3.64, %Cl 2.66.

Loading of the cellulose plate with triazine was calculated using data from elemental analysis. Based on a nitrogen content of 2.60 mmol (N)/1 g, nitrogen loading (NLw) was calculated as equivalent to NLw = 0.87 mmol (triazine)/1 g. Surface loading (NLs) was calculated based on the nitrogen content as 31.9 × 10^−6^ mol (N)/cm^2^. This was equivalent to NLs= 10.6 × 10^−6^ mol (triazine)/cm^2^. Based on chlorine content, loading (ClLw) was calculated as ClLw= 0.75 mmol (Cl)/1 g, which was equivalent to chlorine surface loading (ClLs) = 9.2 × 10^−6^ mol (Cl)/cm^2^.

### 2.2. Immobilization of M-Fenylenediamine

The 158 plates of paper with attached DCMT were submerged in 1 M solution of *m*-phenylenediamine (Sigma-Aldrich) (43 g) in THF (400 mL) and gently shaken for 24 h at room temperature. The plates were then removed from the *m*-phenylenediamine solution, drained with dry filter paper and washed with THF (2 × 200 mL), N,N-dimethylformamide (DMF) (Baker) (2 × 200 mL) and again with THF (2 × 200 mL) and dried in a vacuum desiccator. The plates were next submerged for 30 min in 25% triethylamine (Et_3_N) (Sigma-Aldrich) solution (250 mL). The excess solution was removed and the sheets were washed twice with DCM (250 mL). 

### 2.3. Loading of C-Terminal Amino Acid. Sub-Libraries {**E**},{**S**} and {H}

For sub-library {**E**}, 52 plates were marked using a graphite pencil with an E. For sub-library {**S**}, 52 plates were marked with an S and for sub-library {**H**} 52 plates were marked with an H. The Fmoc-protected amino acid (25 mmol) and 4-(4,6-dimethoxy-1,3,5-triazin-2-yl)-4-methyl-morpholinium p-toluenosulphonate (DMT/NMM/TosO^−^) (10.32 g, 25 mmol) was dissolved in DMF (65 mL) and then *N*-methylmorpholine (NMM) (5.5 mL, 50 mmol) was added. The solution was left for 5 min to carry out the pre-activation process (for typical method of coupling Fmoc-protected amino acid with amine according solid phase peptide synthesis see ref. 43). For each Fmoc-protected amino acid, functionalized cellulose plates were immersed in the mixture of reagents and shaken gently for 24 h. The excess acylating reagent was removed, then the plates were successively washed by gentle shaking in DMF (3 × 100 mL) and DCM (3 × 50 mL) and dried in a vacuum desiccator. The same procedure was used to prepare sub-library {**E**} with Fmoc-Glu(tBu)-OH (10.65 g, 25 mmol), sub-library {**S**} with Fmoc-Ser(tBu)-OH (9.6 g, 25 mmol) and sub-library {**H**} with Fmoc-His(Trt)-OH (15.5 g, 25 mmol).

### 2.4. Removal of Fmoc-Protecting Group

Each of the sub-libraries {**E**}, {**S**} and {**H**} were treated with 25% solution of piperidine (Sigma-Aldrich) in DMF (400 mL) and shaken gently for 20 min. They were then washed with DMF (3 × 200 mL) and DCM (1 × 200 mL) and used in the next stage of synthesis. 

### 2.5. Incorporation of the Second Amino Acid

Each of the 48 plates of sub-library {**E**} were marked as E,E, E,S or E,H and then immersed in a solution of Fmoc-Glu(tBu)-OH (4.26 g, 10 mmol), to which solution of DMT/NMM/TosO^−^ (4.13 g, 10 mmol) in DMF (50 mL) and NMM (2.2 mL, 20 mmol) were added after pre-activation step. The cellulose plates were shaken gently for 24 hours. The excess acylating reagent was removed, then the plates were washed with gentle shaking in DMF (3 × 100 mL) and DCM (3 × 100 mL) successively. The plates were dried in a vacuum desiccator affording to sub-libraries {**E,E**}, {**E,S**} and {**E,H**}. This standard procedure was repeated with Fmoc-Ser(tBu)-OH (3.84 g, 10 mmol) on 48 plates from sub-library {**S**}, marked S,E, S,S and S,H, to obtain sub-libraries {**S,E**}, {**S,S**} and {**S,H**}. Sub-libraries {**H,S**}, {**H,E**} and {**H,H**} were prepared by marking 48 plates from sub-library {**H**} with a graphite pencil as H,E, H,S and H,H and treating them with Fmoc-His(Trt)-OH (6.2 g, 10 mmol).

### 2.6. Removal of Fmoc-Protecting Group

Each sub-library of dipeptides was immersed in 25% solution of piperidine in DMF (400 mL) and shaken gently for 20 min. It was then washed with DMF (3 × 200 mL) followed by DCM (1 × 200 mL). The sub-libraries were used immediately in the next stage of synthesis.

### 2.7. Incorporation of the N-Terminal Amino Acid

Four copies of nine plates from each of the sub-libraries of dipeptides (36 plates) were marked as S,S,S, E,S,S, H,S,S, S,S,E, E,S,E, H,S,E, S,S,H, E,S,H, H,S,H, S,E,S, E,E,S, H,E,S, S,E,E, E,E,E, H,E,E, S,E,H, E,E,H, H,E,H, S,H,S, E,H,S, H,H,S, S,H,E, E,H,E, H,H,E, S,H,H, E,H,H, H,H,H. These plates were immersed in a solution containing Fmoc-protected amino acid (10 mmol), DMT/NMM/TosO^-^ (4.13 g, 10 mmol) in DMF (50 mL) and NMM (2.2 mL, 20 mmol) (after 5 min. reactivation step). The cellulose plates were shaken gently for 24 h. The excess of acylating reagent was removed, following which the plates were washed first in DMF (3 × 100 mL) with gentle shaking and then in DCM (3 × 100 mL). They were then dried in a vacuum desiccator. This procedure was repeated using Fmoc-Glu(tBu)-OH (4.26 g, 10 mmol), yielding 4 copies of the sub-libraries: {**E,E,E**}, {**E,S,E**}, {**E,H,E**}, {**S,E,E**}, {**S,S,E**}, {**S,H,E**}, {**H,S,E**}, {**H,E,E**}, {**H,H,E**}. Using Fmoc-Ser(tBu)-OH (3.84 g, 10 mmol), 4 more copies of the sub-libraries were obtained: {**S,S,S**}, {**S,E,S**}, {**S,H,S**}, {**S,S,E**}, {**S,E,E**}, {**S,H,E**}, {**S,S,H**}, {**S,E,H**}, {**S,H,H**}. Finally, 4 copies of the sub-libraries were prepared using Fmoc-His(Trt)-OH (6.2 g, 10 mmol): {**H,S,S**}, {**H,E,S**}, {**H,H,S**}, {**H,S,E**}, {**H,E,E**}, {**H,H,E**}, {**H,S,H**}, {**H,E,H**}, {**H,H,H**}.

### 2.8. Removal of Fmoc-Protecting Group

The sub-libraries with immobilized amino acids, dipeptides and tripeptides were immersed in a 25% solution of piperidine in DMF (400 mL) and shaken gently for 20 minutes. They were then washed, first with DMF (3 × 200 mL) and then in DCM (1 × 200 mL), before being used immediately in the next stage of synthesis.

### 2.9. Incorporation of Lipid Fragment

Sets from the sub-libraries of amino acids **3**, dipeptides **6** and tripeptides **8** were additionally marked with **a** (for heptanoic acid) (Sigma-Aldrich), **b** (for octanoic acid) (Sigma-Aldrich), **c** (for (E)-octadec-9-enoic (elaidic) acid) (Sigma-Aldrich) or **d** (for hexadecanoic acid) (Sigma-Aldrich). A solution of DMT/NMM/TosO^−^ (12.4 g, 30 mmol) in DCM (100 mL) was cooled to 0−5 °C and treated with carboxylic (fatty) acid **4a–d** (30 mmol) and NMM (0.85 mL, 7.5 mmol). Stirring was continued at 0–5 °C for 4 hours to form in situ a triazine fatty acid ester (for more details see ref. 39). The plates were immersed in the solution of obtained triazine fatty acid esters and shaken gently at room temperature for 24 hours. All the plates were then soaked and washed successively in DCM (4 × 200 mL), DMF (2 × 200 mL) and DCM (2 × 200 mL).

Acylation was performed with heptanoic acid (**4a**) (3.9 g, 30 mmol), yielding: {**5 a,E**}, {**5 a,S**}, {**5 a,H**}, {**7 a,E,E**}, {**7 a,S,E**}, {**7 a,H,E**}, {**7 a,E,S**}, {**7 a,S,S**}, {**7 a,H,S**}, {**7 a,E,H**}, {**7 a,S,H**}, {**7 a,H,H**}, {**9 a,E,E,E**}, {**9 a,S,E,E**}, {**9 a,H,E,E**}, {**9 a,E,S,E**}, {**9 a,S,E,S**}, {**9 a,H,E,S**}, {**9 a,E,H,E**}, {**9 a,S,E,H**}, {**9 a,H,E,H**}, {**9 a,E,S,E**}, {**9 a,S,E,S**}, {**9 a,H,S,E**}, {**9 a,E,S,S**}, {**9 a,S,S,S**}, {**9 a,H,S,S**}, {**9 a,E,S,H**}, {**9 a,S,H,S**}, {**9 a,H,S,H**}, {**9 a,E,H,E**}, {**9 a,S,H,E**}, {**9 a,H,E,H**}, {**9 a,E,H,S**}, {**9 a,S,H,S**}, {**9 a,H,S,H**}, {**9 a,E,H,H**}, {**9 a,S,H,H**}, {**9 a,H,H,H**}.

Acylation using octanoic acid (**4b**) (5.16 g, 30 mmol) yielded: {**5 b,E**}, {**5 b,S**}, {**5 b,H**}, {**7 b,E,E**}, {**7 b,S,E**}, {**7 b,H,E**}, {**7 b,E,S**}, {**7 b,S,S**}, {**7 b,H,S**}, {**7 b,E,H**}, {**7 b,S,H**}, {**7 b,H,H**}, {**9 b,E,E,E**}, {**9 b,S,E,E**}, {**9 b,H,E,E**}, {**9 b,E,S,E**}, {**9 b,S,E,S**}, {**9 b,H,E,S**}, {**9 b,E,H,E**}, {**9 b,S,E,H**}, {**9 b,H,E,H**}, {**9 b,E,S,E**}, {**9 b,S,E,S**}, {**9 b,H,S,E**}, {**9 b,E,S,S**}, {**9 b,S,S,S**}, {**9 b,H,S,S**}, {**9 b,E,S,H**}, {**9 b,S,H,S**}, {**9 b,H,S,H**}, {**9 b,E,H,E**}, {**9 b,S,H,E**}, {**9 b,H,E,H**}, {**9 b,E,H,S**}, {**9 b,S,H,S**}, {**9 b,H,S,H**}, {**9 b,E,H,H**}, {**9 b,S,H,H**}, {**9 b,H,H,H**}.

Acylation using (E)-octadec-9-enoic (elaidic) acid (**4c**) (8.47 g, 30 mmol) afforded: {**5 c,E**}, {**5 c,S**}, {**5 c,H**}, {**7 c,E,E**}, {**7 c,S,E**}, {**7 c,H,E**}, {**7 c,E,S**}, {**7 c,S,S**}, {**7 c,H,S**}, {**7 c,E,H**}, {**7 c,S,H**}, {**7 c,H,H**}, {**9 c,E,E,E**}, {**9 c,S,E,E**}, {**9 c,H,E,E**}, {**9 c,E,S,E**}, {**9 c,S,E,S**}, {**9 c,H,E,S**}, {**9 c,E,H,E**}, {**9 c,S,E,H**}, {**9 c,H,E,H**}, {**9 c,E,S,E**}, {**9 c,S,E,S**}, {**9 c,H,S,E**}, {**9 c,E,S,S**}, {**9 c,S,S,S**}, {**9 c,H,S,S**}, {**9 c,E,S,H**}, {**9 c,S,H,S**}, {**9 c,H,S,H**}, {**9 c,E,H,E**}, {**9 c,S,H,E**}, {**9 c,H,E,H**}, {**9 c,E,H,S**}, {**9 c,S,H,S**}, {**9 c,H,S,H**}, {**9 c,E,H,H**}, {**9 c,S,H,H**}, {**9 c,H,H,H**}.

Acylation using hexadecanoic acid (**4d**) (7.7 g, 30 mmol) yielded: {**5 d,E**}, {**5 d,S**}, {**5 d,H**}, {**7 d,E,E**}, {**7 d,S,E**}, {**7 d,H,E**}, {**7 d,E,S**}, {**7 d,S,S**}, {**7 d,H,S**}, {**7 d,E,H**}, {**7 d,S,H**}, {**7 d,H,H**}, {**9 d,E,E,E**}, {**9 d,S,E,E**}, {**9 d,H,E,E**}, {**9 d,E,S,E**}, {**9 d,S,E,S**}, {**9 d,H,E,S**}, {**9 d,E,H,E**}, {**9 d,S,E,H**}, {**9 d,H,E,H**}, {**9 d,E,S,E**}, {**9 d,S,E,S**}, {**9 d,H,S,E**}, {**9 d,E,S,S**}, {**9 d,S,S,S**}, {**9 d,H,S,S**}, {**9 d,E,S,H**}, {**9 d,S,H,S**}, {**9 d,H,S,H**}, {**9 d,E,H,E**}, {**9 d,S,H,E**}, {**9 d,H,E,H**}, {**9 d,E,H,S**}, {**9 d,S,H,S**}, {**9 d,H,S,H**}, {**9 d,E,H,H**}, {**9 d,S,H,H**}, {**9 d,H,H,H**}.

### 2.10. Removal of Side Chain Protecting Groups

All of the functionalized cellulose plates were treated with 400 mL solution of TFA in DCM (50% v:v) in the presence of triispropylsilane (TIS) (2% v:v) (Sigma-Aldrich) and water (3% v:v) for 2 hours. The plates were then washed with DCM (2 × 200 mL), ethanol (EtOH) (2 × 200 mL) (Avantor) and DCM (2 × 200 mL) and dried in a desiccator. For subsequent hydrolysis experiments, all the sheets were divided into small strips (3 × 16 mm). Each strip was individually marked.

### 2.11. Synthesis of Z-Leu-NA (10)

To DMT/NMM/TosO^−^ (2.06 g, 5.0 mmol) in DCM (30 mL), cooled in an ice-water bath solution with intense stirring, was added Z-Leu-OH (1.33 g, 5.0 mmol). After 15 min, NMM (0.27 mL, 2.5 mmol) was added. Once the reaction was complete, as determined by TLC (DMT/NMM/TosO^−^ spot disappearance, NBP visualization) (about 1 h), *p*-nitroaniline (0.69 g, 5.0 mmol) and a catalytic amount of DMAP (2 mg) were added. Stirring was continued for 12 h. The mixture was washed with water (30 mL), 1M NaHSO_4_ (30 mL) and again with water (30 mL). The organic layer was dried over MgSO_4_, the drying agent was filtered off and the filtrate was concentrated on a vacuum evaporator, affording 1.11 g Z-Leu-NA (**10**) with 58% yield. The residue was dried to constant weight in a vacuum desiccator over P_2_O_5_ and KOH. Anal. RP-HPLC (3–97% B in 30 min): t_R_ 22.81 min, purity 98%. M.p. 127–129 °C; lit. [42] 128–129 °C.

^1^H-NMR (250 MHz, CDCl_3_): δ = 0.91 (6H, J = 6.6 Hz, (CH_3_)_2_–CH–); 1.34–1.56 (m, 3H, –CH_2_CH–); 4.43 (dt, 1H, J_1_ = 8.1 Hz, J_2_ = 4.5 Hz, –CH_2_CH–); 5.01 (bs, 2H, –CH_2_O–); 7.24–7.33 (m, 5H, arom); 7.35 (dd, 2H, J_1_ = 8.66 H, J_2_ = 2.8 Hz, arom.); 8.13 (dd, 2H, J_1_ = 8.66 H, J_2_ = 2.8 Hz, arom.) [ppm]. ^13^C–NMR (75 MHz, CDCl_3_): δ = 22.6; 24.8; 40.4; 53.6; 117.3; 118.8; 127.4; 128.5; 129.1; 155.8; 170.8 [ppm].

### 2.12. Hydrolysis of Z-Leu-NA in the Presence of N-Lipidated Amino Acids, Di- and Tripeptides Immobilized on Cellulose

Strips of equal size (16 mm × 3 mm) were taken from the cellulose plates functionalized with *N*-lipidated amino acids (**5**), dipeptides (**7**) and tripeptides (**9**). These were treated with 50 mL phosphate buffer pH = 7 (50 mL) for 30 min and then washed twice with water (50 ml) and 50 mL water-methanol solution (50%:50%) (v:v). The strips were then dried to a constant mass. Finally, they were fastened round the side walls of ELISA plate wells, with care being taken to avoid disturbing light transmission. 

#### 2.12.1. Hydrolysis of Z-Leu-NA in Solution at pH 7

A solution of Z-Leu-NA at 0.1 M concentration in a mixture of water/methanol (20% v:v, 150 μL) was added to each well in the ELISA plate. Extinction was measured at 405 nm with a 120 s time interval using a Sunrise-Tecan (Männedorf, Switzerland) microplate reader at 22 °C. The collected data were processed with Excel. 

#### 2.12.2. Hydrolysis of Z-Leu-NA in Solution at pH 8.5

A solution of Z-Leu-NA at 0.1 M concentration in a mixture of water (20 µL), NMM (45 µL) and methanol (95 µL) (12% H_2_O, 28 % NMM, 60 % MeOH (v:v:v, 160 µL) was added to each plate well. Extinction was measured at 405 nm with a 120 s time interval using a Sunrise-Tecan microplate reader at 22 °C. The collected data were processed with Excel. 

### 2.13. Docking of Metal Iions into the Catalytic Pockets of a Synzymes Library Immobilized on Cellulose

Buffered strips, dried to constant mass, were treated with relevant solutions of metal salts at a concentration of 0.01 mmol/mL, prepared by dissolving 0.5 mmol CuCl_2_·2H_2_O (82 mg) in 50 mL H_2_O or 0.5 mmol ZnCl_2_ (68 mg) in 50 mL H_2_O. After 1 h, the excess salt solution was removed and the modified cellulose strips were washed with water. The strips were again dried to a constant mass.

### 2.14. Hydrolysis of Z-Leu-NA in the Presence of N-Lipidated Amino Acids, Di- and Tripeptides Immobilized on Cellulose and Metal Ions

The progress of Z-Leu-NA hydrolysis in the presence of metal ions was measured as described above in 2.12.2.

### 2.15. Blank Experiments

In all experiments as a blanks were used: unmodified cellulose; cellulose acylated with hexadecanoic, octadec-9-enoic and heptanoic acids. In the presence of blanks, Z-Leu-NA was stable. Observed value of absorbance was lower than 0.1. 

## 3. Results and Discussion

**Figure 1:**
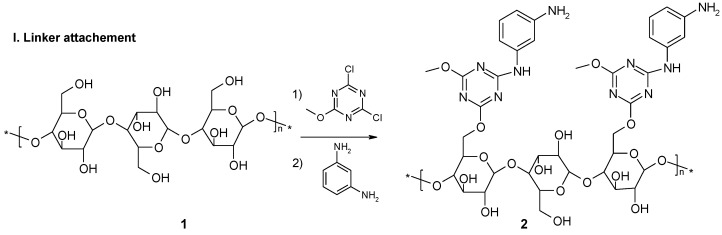

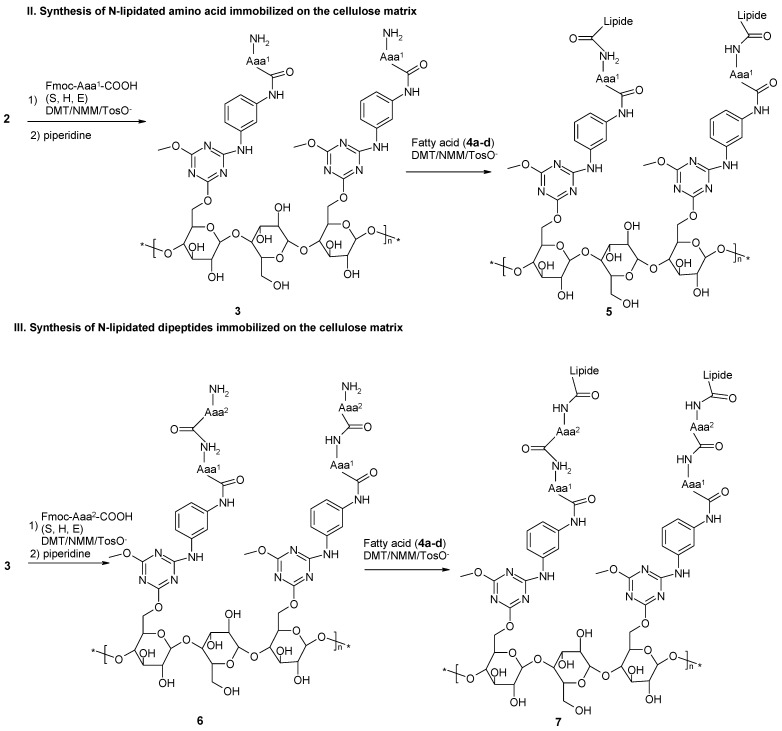

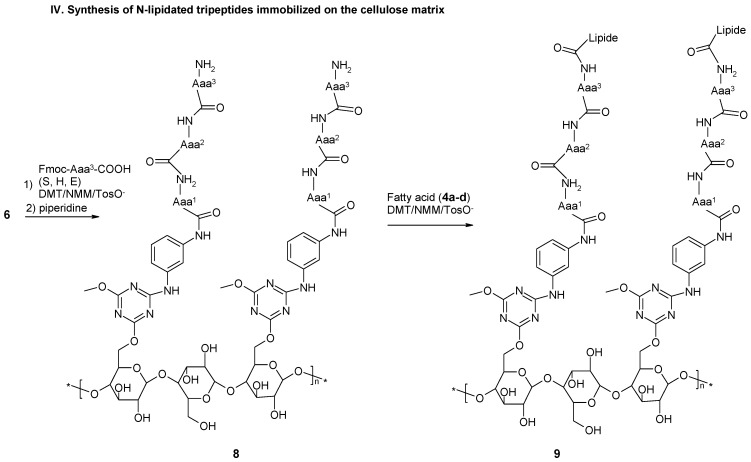
Synthesis of *N*-lipidated amino-acids **5**, dipeptides **7** and tripeptides **9** immobilized on cellulose.

The peptide fragments were attached to the cellulose **1** by the relatively rigid bi-aromatic linker [39]. The rings, besides providing good exposure for the peptide fragments, facilitated π-acceptor - π-donor interactions and ensured proton acceptor properties, due to the presence of the basic triazine ring in each binding pocket. The linker was formed by treating the cellulose (**1**) with DCMT and subsequently with *m*-phenylenediamine (see Figure 1, step I). 

**Figure 2:**
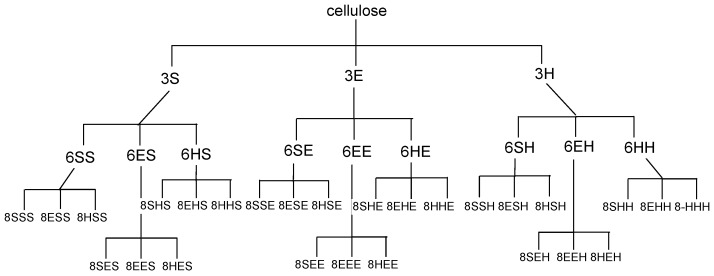
Diagram showing 39 precursors **3**, **6**, **8**, which after *N*-acylation with four carboxylic acids **4a-d** yielded a 156 element library of synzymes **5**, **7** and **9**.

For synthesis of the peptide fragment, 3 amino acids which occur in the catalytic triad of many hydrolases: histidine, serine and glutamic acid were used. In natural enzymes, aspartic acid is more commonly found than glutamic acid. However, it was assumed that the more flexible Glu side chain, due to the presence of an additional methylene group in the catalytic cavity, would be advantageous for interactions with substrates of various shapes. Peptide synthesis on a modified cellulose support **2** proceeded according to the Fmoc/But strategy using DMT/NMM/TosO^−^ as the coupling reagent [43]. After the completion of each coupling step, the Fmoc group was cleaved with piperidine. The synthesis gave 12 plates with an attached single amino-acid residue **5**, 36 plates with dipeptides **7** and 108 plates with tripeptides **9**. Each structure **3**, **6**, **8** was prepared in 4 identical copies. Each of these copies was acylated with one of four fatty acids **4a–d**, pre-activated before coupling with DMT/NMM/TosO^-^ to obtain the appropriate triazine superactive ester [44]. This procedure yielded a library of 156 lipidated peptides/amino-acids **5**, **7**, **9**. The last stage of synthesis involved the removal of the protecting groups from the amino acid functional groups in the side chains, using trifluoroacetic acid. The 39 amino-acid/peptide fragments used for the synthesis of the 156 element library are presented in Figure 2.

This study set out to investigate:

1. whether structures composed of S, H and E residues would promote the hydrolysis of the amide/peptide bond (protease activity),
2. the optimal synzyme structure for hydrolysis of the amide/peptide bond,
3. if the synzymes could be used many times, giving the possibility of their use in many tests.

**Figure 3:**
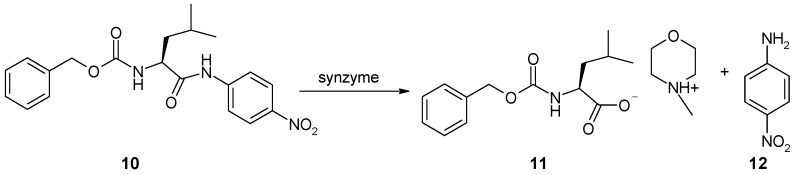
Hydrolysis of Z-Leu-NA (**10**) in the presence of synzyme leading to the formation of salt of Z-Leu-OH with NMM (Z-Leu-O^−^ HNMM^+^, **11**) and *p*-nitroaniline (**12**).

The hydrolytic activity of structures **5**, **7** and **9** was tested using Z-Leu-NA (**10**) as a substrate. Hydrolysis was monitored by measuring the absorption characteristics for *p*-nitroaniline at 405 nm. In preliminary experiments, identically-sized strips of functionalized cellulose **5**, **7**, **9** were treated with phosphate buffer (pH = 7), mounted around the walls of ELISA microplate wells and then submerged in 0.1 M methanolic solution of Z-Leu-NA containing 10% water (v:v). It was found that hydrolysis did not occur under these conditions. Raising the temperature of the reaction from room temperature to 30 °C also had no effect. Therefore, it was necessary to find optimal conditions for Z-Leu-NA hydrolysis in the presence of the synthetized synzymes. Hydrolysis of the amide bond is known to occur more easily in a slightly alkaline environment. Therefore, an attempt was made to dilute a methanolic solution of Z-Leu-NA with aqueous solution of phosphate buffer to pH 8 or pH 9. Unfortunately, the addition of aqueous solutions caused precipitation of the substrate, which made it impossible to carry out spectrophotometric tests. This problem was solved by using *N*-methylmorpholine as the base, which ensured a solution of pH 8.5 with good substrate solubility. Further studies were therefore conducted using 0.1 M solution of Z-Leu-NA in a solvent mixture containing 12% H_2_O, 28% NMM and 60% MeOH (v:v:v). In screening tests, a 4-fold excess of Z-Leu-NA was applied in relation to the theoretical amount of synzyme per unit area of the cellulose strip. Another obstacle was the time-consuming process of filling the wells with the reaction media and arranging the catalytic strips around the walls of the ELISA plate wells, so as to avoid loosed strips from impeding light transmission. To standardize the experimental conditions, collection of spectrophotometric data was started 10 min after the first row of wells had been filled with substrate solution. This practice privileged uniform measurement conditions after 10 min of pre-activation, at the price of missing data from the early stage of hydrolysis (Figure 3).

**Figure 4:**
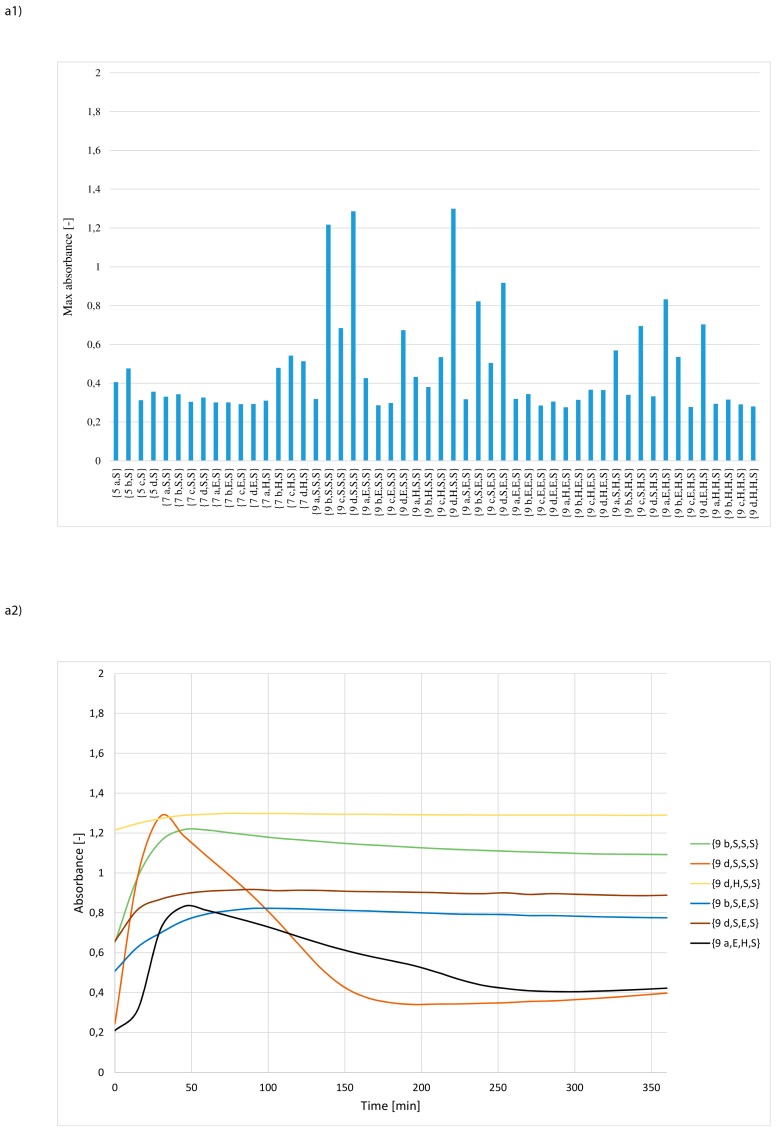

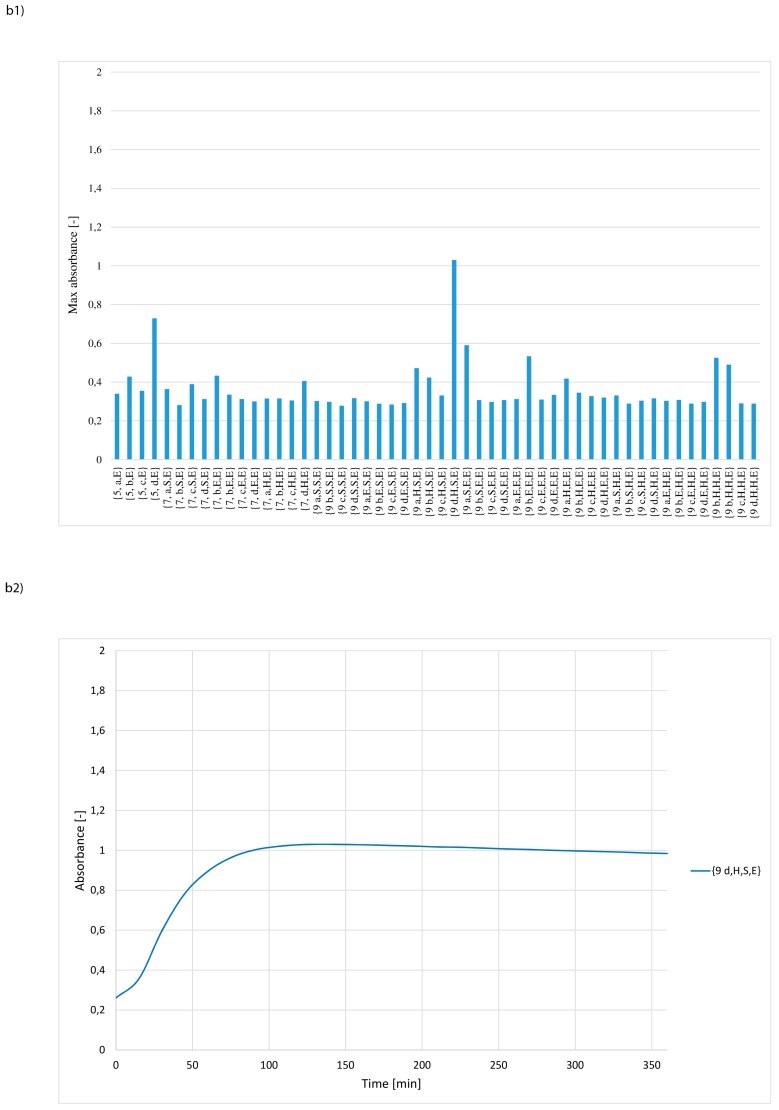

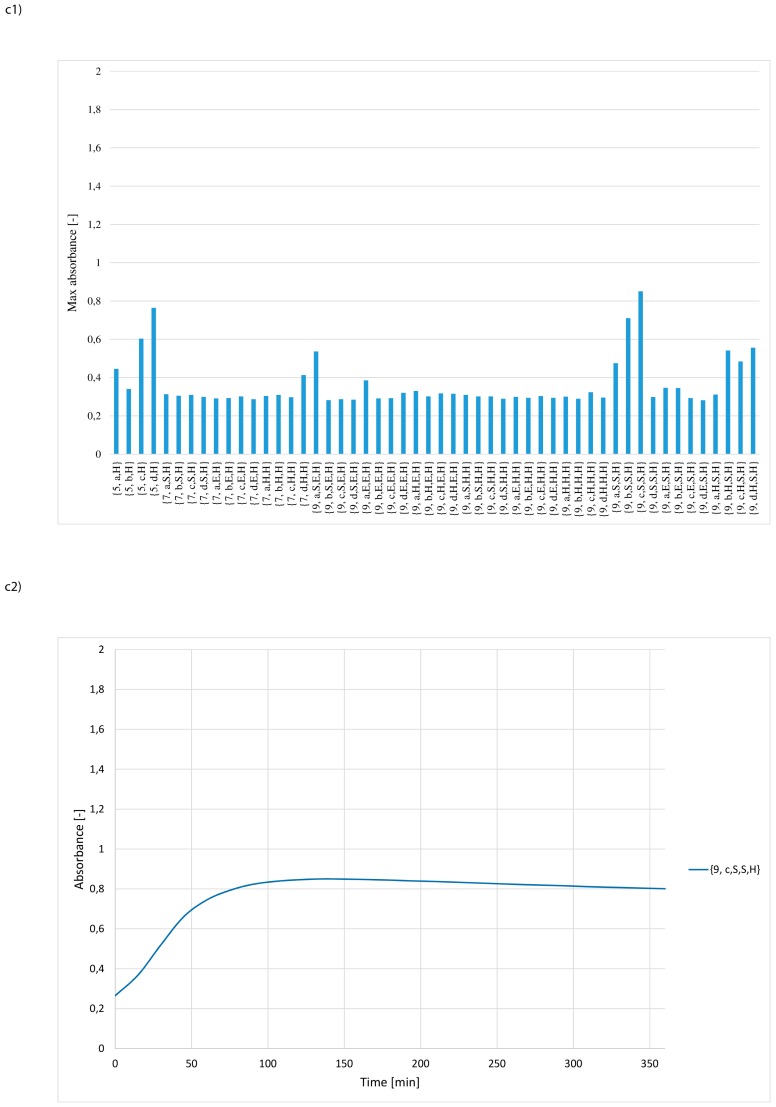
Selection of the most active synzymes (maximum absorbance assay) (upper panel 1) and measurement of Z-Leu-NA hydrolysis in a solution at pH = 8.5 using the selected the most active synzymes (lower panel 2): (**a**) sub-library of synzymes with serine reside at C-terminal positions, (**b**) sub-library of synzymes with glutamic acid reside at C-terminal positions, (**c**) sub-library of synzymes with histidine reside at C-terminal positions.

The results of 156 independent measurements are depicted in Figure 4a−c and in Figure S1 in Supporting Materials. In order to select catalytically most active structures, the criterion of maximum absorbance was applied. It has been assumed that those synzymes for which the maximum absorbance value was at least 0.8 can be considered as sufficiently effective catalysts. The criterion met only 8 derivatives, which represented less than 6% of the entire library. This group included only *N*-lipidated tripeptides: {**9 b,S,S,S**}, {**9 d,S,S,S**}, {**9 d,H,S,S**}, {**9 b,S,E,S**}, {**9 d,S,E,S**}, {**9 a,E,H,S**}, {**9 d,H,S,E**} and {**9 c,S,S,H**}, 6 selected derivatives contained the serine residue at the C-terminal position. Analysing the structure of the selected synzymes it could be noted that the structure of the fatty acid in the tripeptides is not a factor affecting the efficiency of hydrolysis. For the other synzymes, max. absorbance was relatively low. This can be attributed to non-active or poorly active catalytic structures.

For the selected 8 synzymes, the dependences between the absorbance and time of Z-Leu-NA hydrolysis were presented (Figure 4a2,b2,c2). The analysis of the curves indicated the existence of four independent cases. In the first group of catalysts the absorbance steadily increased during the hydrolysis progress until the plateau was reached. This pattern was observed for structures: {**9 b,S,S,S**}, {**9 b,S,E,S**}, {**9 d,S,E,S**}, {**9 d,H,S,E**} and {**9 c,S,S,H**}. In the second group, a rapid increase of absorbance in the early phase of hydrolysis was followed by a very gradual decrease. This may have been caused by clogging of the catalytic pocket by the reaction product, followed by its precipitation or by the self-digestion of amide bonds in the peptide fragment of the catalyst. The least frequent curve (occurring only in case of **9 d,S,S,S** and **9 a,E,H,S,** Figure 4a2) shows a very rapid increase in absorbance, which then falls sharply and subsequently stabilizes at a level typical for catalysts of the second group. In this case as well as for some structures belonging to the second group, experimental examination of the ELISA plate wells revealed the formation of turbidity and a solid deposit in the medium. The last case is the curve obtained using **9 d,H,S,S**, where in the initial time a very intense increase in absorbance was observed, which after fast reaching the maximum value during the progress of hydrolysis did not change. 

It was found that the all active synzymes **9** contained tripeptides in the catalytic pocket and were serine rich. In 6 cases, serine residues were dominated in the peptide fragment and in two cases serine residues were present exclusively in the most active catalytic structure. This suggests that the increased size and highly polar characteristic of the binding pockets had a very important effect on catalytic activity. Given the lipophilic nature of substrate **10**, it also means that binding of the substrate is not a limiting factor in the catalytic process. The presence of a plateau or even a decrease in absorption after a relatively fast rate of hydrolysis in the preliminary stage suggests that the diffusion of slightly soluble *p*-nitroaniline from the catalytic pocket may have had a strong impact on transformation. 

An attempt to directly correlate the research results with literature data is quite difficult. This is due to the incompatibility of catalytic structures with other synzymes with peptidic active fragment [44,45,46,47,48,49,50]. The comparison of the effectiveness of *N*-lipidated peptides immobilized on cellulose to catalyse the hydrolysis of esters [40,41] and amides indicates that degradation of amide/peptide bond is more demanding and possible only with highly active catalytic systems. Hydrolysis of *p*-nitrophenyl esters of *N*-protected amino acids occurred very effectively in the presence of the most of structures composed of serine, histidine and glutamic acid. Thus, in order to diversify the esterase catalytic activity of the *N*-lipidated peptides, it was necessary to use highly hindered *p*-nitrophenyl esters such as Z-Ala-Aib-ONp or Z-Aib-Aib-ONp (Aib – α-metylalanine). 

**Figure 5:**
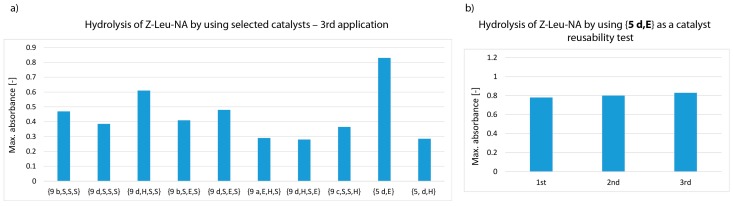
Measurement of Z-Leu-NA hydrolysis using the 9 most effective synzymes at pH 8.5 (third catalytic cycle, left panel a). Re-usability test of **5 d,E** (panel b).

Attempts were made to verify whether the catalytically active synzymes could be exploited repeatedly. A pool of the 8 most active structures selected in the first screening and also the 2 most active structures with single amino acid residue, {**5 d,E**} and {**5 d,H**} with max absorbance above 0.75 (see Figure 4b1,c1) were applied. Before reuse, the strips were regenerated by thorough washing with methanol to remove any deposits and re-buffered to pH 7. For one catalytic turnover a 4-fold excess of Z-Leu-NA was applied in relation to the calculated amount of *N*-lipidated peptides attached to the cellulose strip used in experiment. This means that after the third cycle the molar ratio substrate/catalytic podands reached value 12:1. It was found that the effectiveness of the synzymes reduced steadily in subsequent catalytic cycles (Figure 5), from absorbance exceeding 0.8 to values to the range of 0.3–0.6, with the exception of {**5 d,E**}. For this catalyst the max. absorbance was stable in reusability test.

In this case the rate of hydrolysis was initially very high but after 45 minutes a slowly drop in the reaction rate was observed (see Supporting Material Figure S2). After completion of the entire measurement, it was found that precipitation had occurred, which was the most probable cause of the decrease in absorbance. No precipitation was observed during the measurements in the third catalytic cycle of the other tested synzymes, which contained three amino acid residues in the active centre. This supports the hypothesis that the product deposits clogging the catalytic pocket were not completely removed in regeneration procedure or that the peptide fragment in the catalytic pockets underwent self-destruction. It has be presumed that in the case of small peptide fragments (with one amino-acid residue), the podants that form the catalytic pocket were too short to interact with each other and auto-destruction was severely limited. 

**Figure 6:**
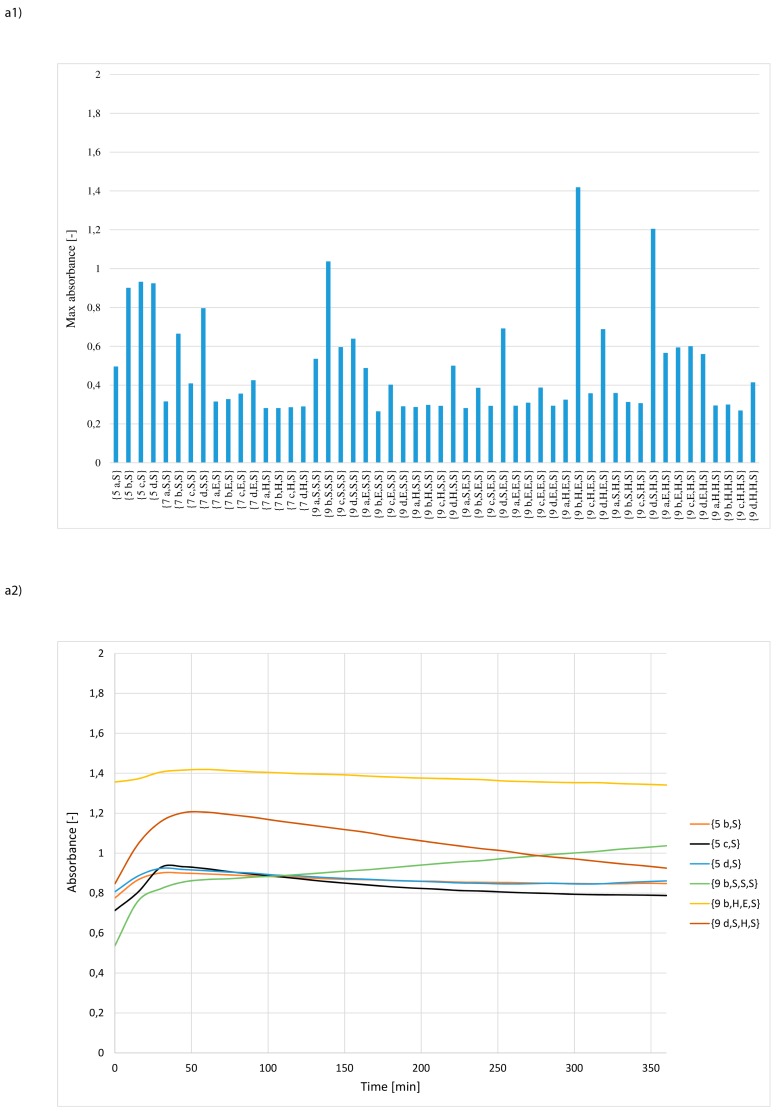

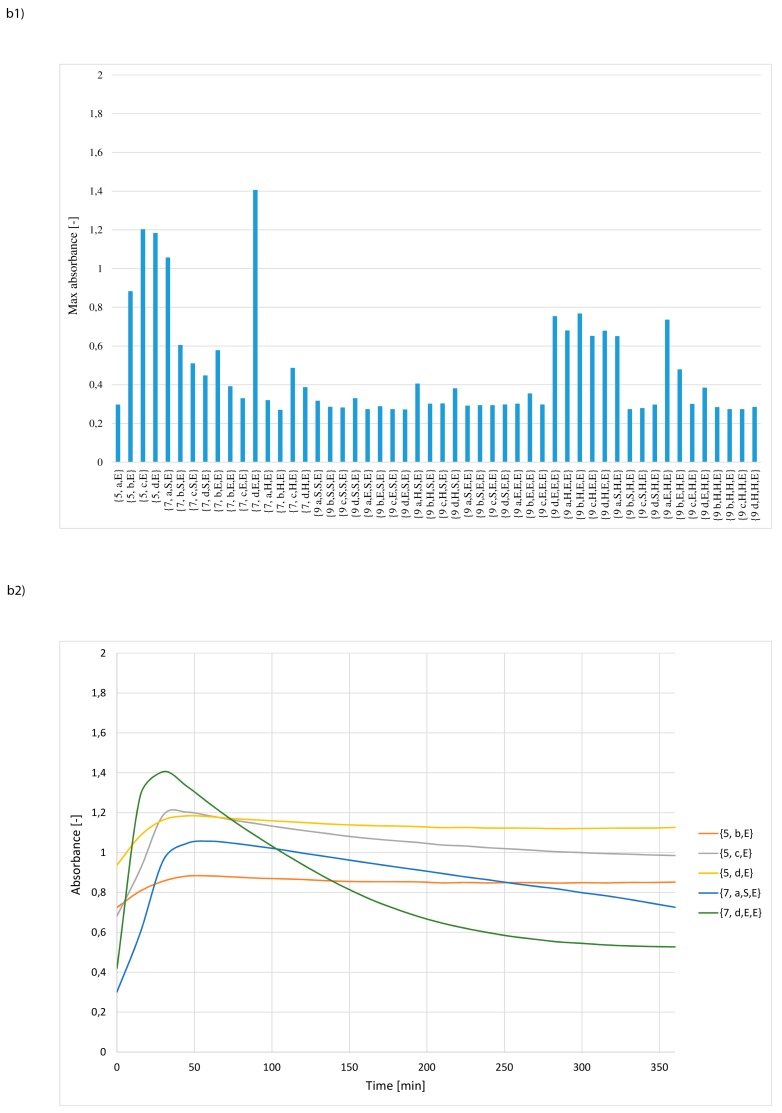

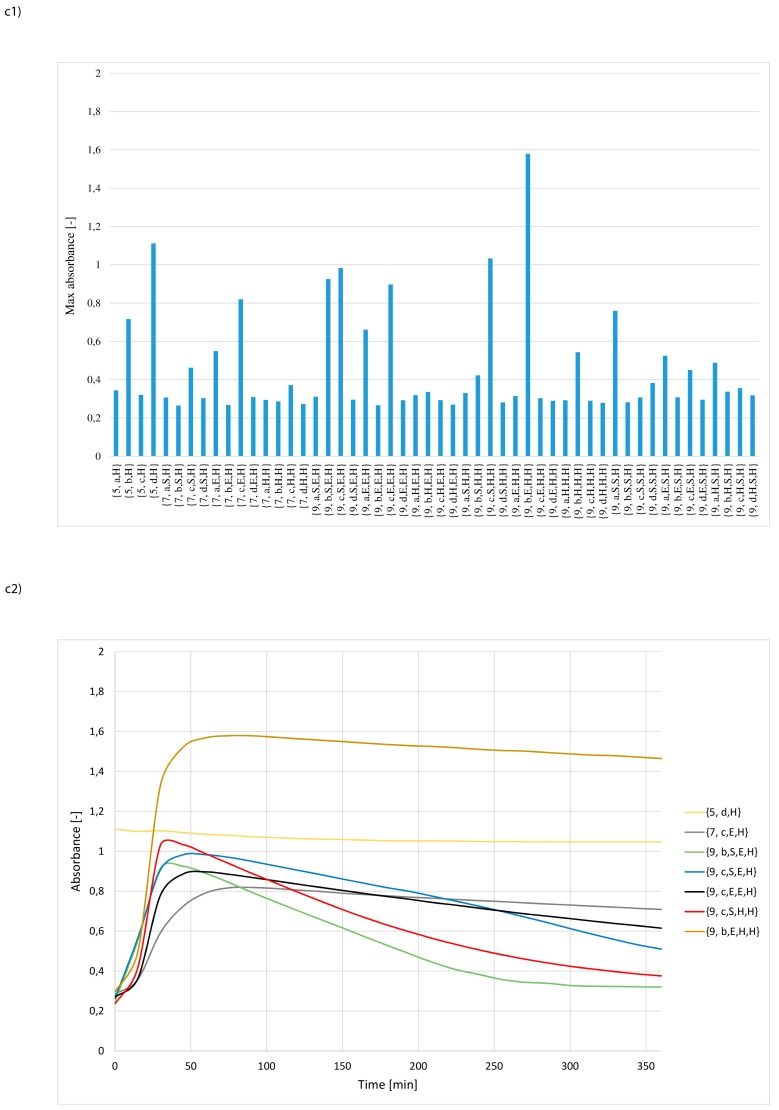
Selection of the most active synzymes (maximum absorbance assay) (upper panel 1) and measurement of Z-Leu-NA hydrolysis in a solution at pH = 8.5 using the selected the most active synzymes (lower panel 2) with Cu^2+^ ions docked in the catalytic pocket: (**a**) sub-library of synzymes with serine reside at C-terminal positions, (**b**) sub-library of synzymes with glutamic acid reside at C-terminal positions, (**c**) sub-library of synzymes with histidine reside at C-terminal positions.

Many natural proteases are known to contain a metal ion in the active centre [51,52]. An example is carboxypeptidase A (CPA), which has peptidase and esterase activity only in the presence of Zn^2+^ ions [53]. Moreover, the activity of CPA can be modulated by exchanging Zn^2+^ with a different metal ion [54]. Esterase activity but not peptidase activity, is observed in the presence of Hg, Cd and Pb ions, whereas with Cu^2+^ there is a complete loss of enzymatic activity by CPA. Therefore, it has been investigated the influence of Cu^2+^ and Zn^2+^ ions. Copper ion were introduced by treating the 156-element library of synzymes with pH = 7 buffer followed by submersion in 0.5 M aqueous CuCl_2_·2H_2_O for 1 h. The excess copper salt was removed by washing with water and finally the strips were dried to a constant weight. The hydrolysis rate was measured under conditions identical to those described above. As in the previous screening, structures for which the absorbance was above 0.8 were considered catalytically active (Figure 6a1,b1,c1 and Figure S3). 

**Figure 7:**
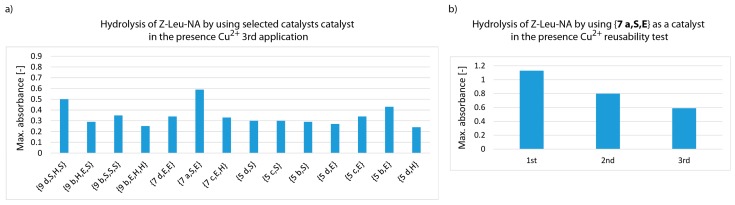
Measurement of Z-Leu-NA hydrolysis at pH 8.5 using the most effective synzymes with Cu^2+^ docked in the active pocket (third catalytic cycle, left panel a). Hydrolytic efficiency of **7 a,S,E** in the presence of Cu^2+^ ions after three subsequent re-use (right panel b).

Generally, the presence of Cu^2+^ ions enhanced catalytic activity. Seven derivatives containing one amino acid residue in the catalytic centre were selected from the library of synzymes: {**5 b,S**}, {**5 c,S**}, {**5 d,S**}, {**5 b,E**}, {**5 c,E**}, {**5 d,E**}, {**5 d,H**}. Three derivatives containing dipeptide in the catalytic centre were chosen: {**7 a,E,E**}, {**7 a,S,E**}, {**7 c,E,H**}. Eight structures with tripeptide were selected: {**9 b,S,S,S**}, {**9 b,H,E,S**}, {**9 d,S,H,S**}, {**9 b,S,E,H**}, {**9 c,S,E,H**}, **{9 c,S,H,H**}, {**9 b,E,H,H**} and {**9 c,E,E,H**}. The criterion (exceeding 0.8 max. absorbance) met 11.5% of catalytically active complexes Cu^2+^-synzyme. In addition, a group of 6 derivatives with moderate activity (absorbance in the range of 0.7–0.8) were chosen from the library. This group included one derivative containing a *N*-lipidated amino acid {**5 b,H**}, one *N*-lipidated dipeptide {**7 d,S,S**} and 4 derivatives containing tripeptide in a catalytic pocket: {**9 d,E,E,E**}, {**9 a,E,H,E**}, {**9 b,H,E,E**} and {**9 a,S,S,H**}. The structures of the most active synzymes in the presence of Cu^2+^ ions were substantially more diverse than those selected in the first screening in the absence of metal ions. The size of the catalytic pocket was not crucial for catalytic activity. As well as serine, histidine and glutamic acid residues predisposed to bind metal ions were also present in the most active structures. It can be noted that only {**9 b,S,S,S**} showed intense catalytic activity in both the presence and absence of Cu^2+^ ions. The analysis of the dependence of absorbance on the reaction time indicated three cases: (1) absorbance gradually increases over time, (2) very rapid increase in absorbance at the initial stage of transformation and reaching the plateau and (3) the initial phase with rapid increase of absorbance followed by its subsequent lowering (Figure 6a2,b2,c2). To test the possibility of re-using the synzymes with docked Cu^2+^ ion, only the pool of the most active derivatives was used. Again, a significant decrease in proteolytic activity was observed after reusing of the catalysts. The most active derivatives after the third catalytic cycle were {**9 d,S,H,S**}, {**7 a,S,E**} and {**5 b,E**} (Figure 7 and Figure S4).

**Figure 8:**
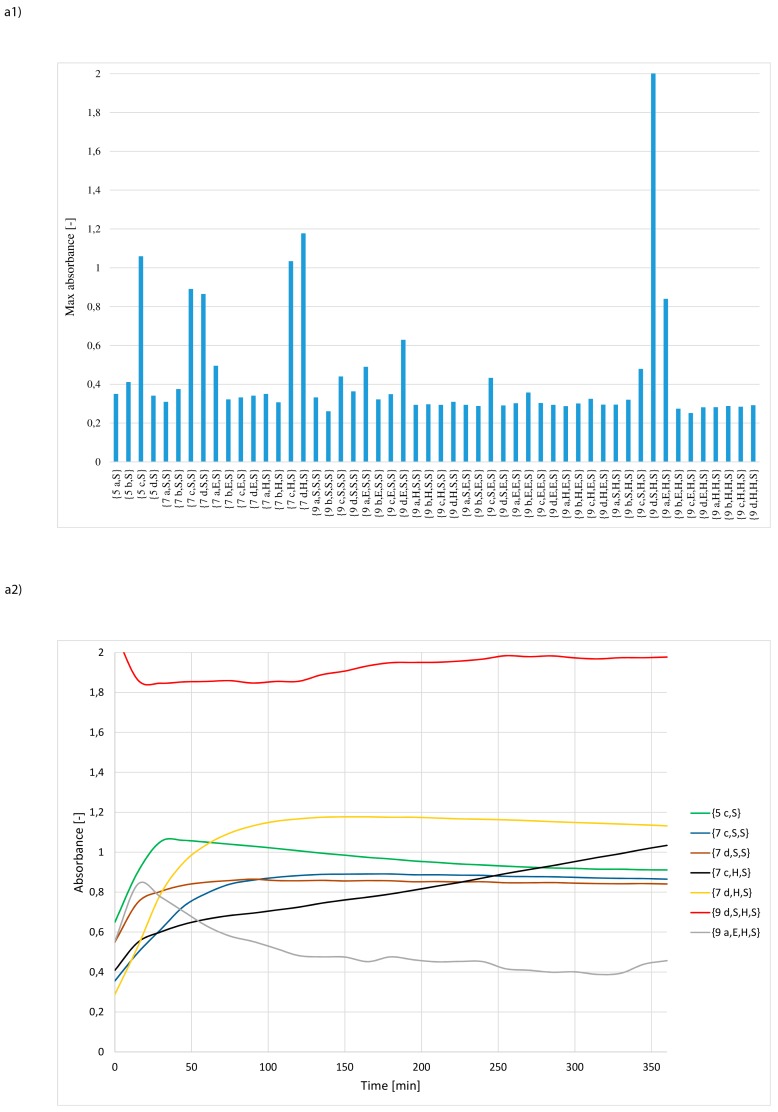

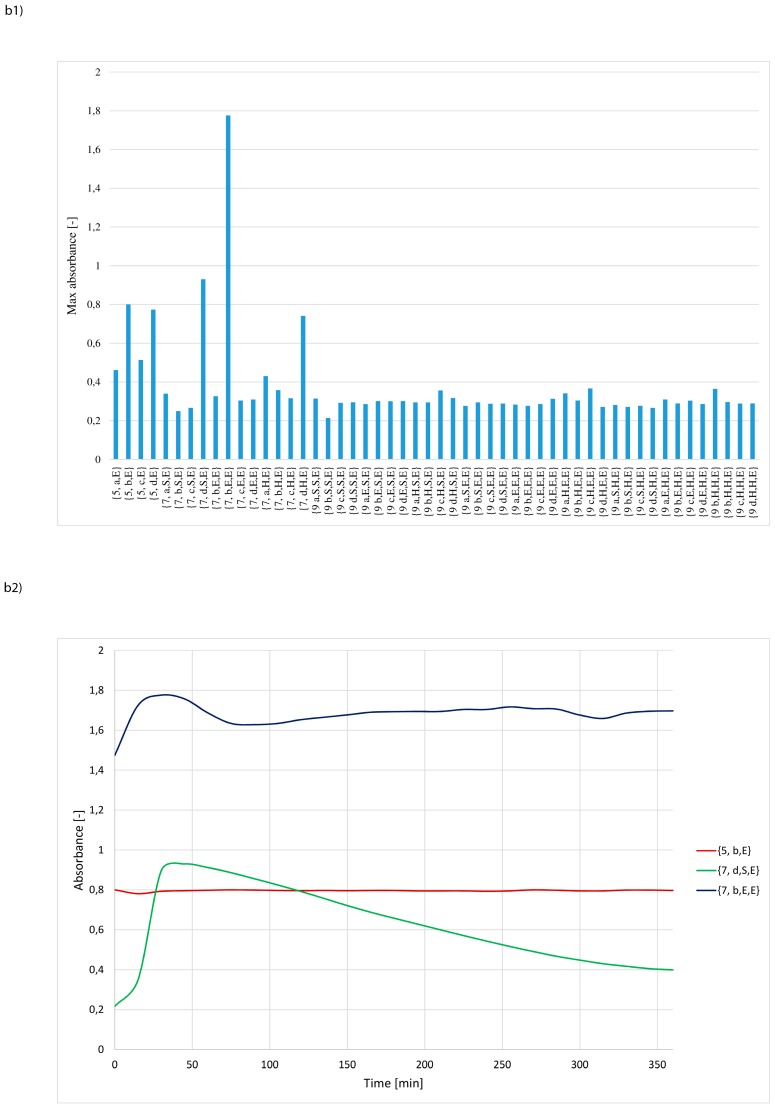

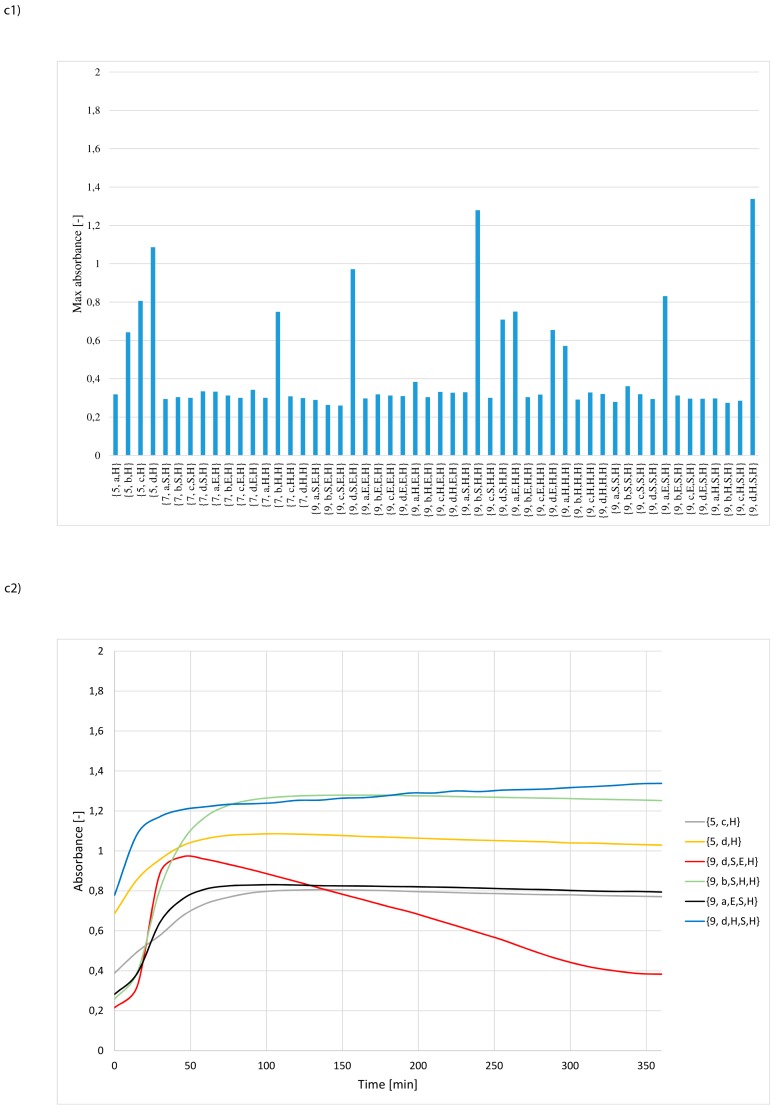
Selection of the most active synzymes (maximum absorbance assay) (upper panel 1) and measurement of Z-Leu-NA hydrolysis in a solution at pH = 8.5 using the selected the most active synzymes (lower panel 2) with Zn^2+^ ions docked in the catalytic pocket: (**a**) sub-library of synzymes with serine reside at C-terminal positions, (**b**) sub-library of synzymes with glutamic acid reside at C-terminal positions, (**c**) sub-library of synzymes with histidine reside at C-terminal positions.

The influence of the Zn^2+^ ion on the rate of hydrolysis was also studied. The method used to prepare 156 strips saturated in 0.5 M ZnCl_2_ solution and monitoring the hydrolysis progress was identical to that described previously for copper ions. The results are collected in Figure 8a–c and Figure S5.

The most catalytically active synzymes, affording absorbance exceeding 0.8, included: four structures containing only one amino acid residue in the active centre {**5 c,S**}, {**5 b,E**}, {**5 d,H**} and {**5 c,H**}; six derivatives with a dipeptide fragment {**7 d,S,S**}, {**7 c,S,S**}, {**7 d,H,S**}, {**7 c,H,S**}, {**7 d,S,E**}, {**7 b,E,E**} and six derivatives containing tripeptide fragments in the active pocket {**9 d,S,H,S**}, {**9 a,E,H,S**}, {**9 d,H,S,H**}, {**9 d,S,E,H**}, **{9 b,S,H,H**}, {**9 a,E,S,H**} (Figure 8a1,b1,c1). In the group with moderate activity (absorbance in the range of 0.7–0.8) were identified: one structure with single amino-acid residue {**5 dE**}; two structures with dipeptide {**7 dH,E**}, {**7 bH,H**} and two structures with tripeptide in the catalytic pocket {**9 aE,H,H**}, {**9 dS,H,H**}. In this case as well, the analysis of the dependence of absorbance on the reaction time indicates three different types of curves (Figure 8a2,b2,c2). 

**Figure 9:**
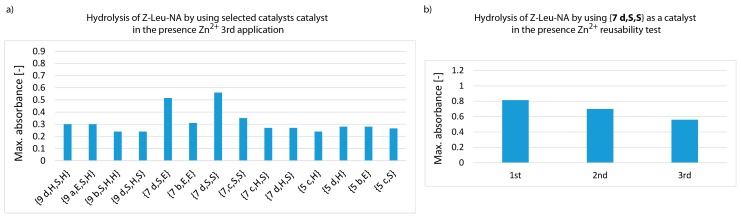
Measurement of Z-Leu-NA hydrolysis at pH 8.5 using the most effective synzymes with Zn^2+^ docked in the active pocket (third catalytic cycle, left panel **a**). Hydrolytic efficiency of **7 d,S,S** in the presence of Zn^2+^ ions after three subsequent re-use (right panel **b**).

In studies on the re-use of the most active structures, a decrease in catalytic activity was observed after each subsequent catalytic cycle. Of all the structures in the tested pool, {**7 d,S,S**} and {**7 d,S,E**} were the most resistant to declining activity (Figure 9 and Figure S6).

The catalytic activity of all the other tested catalysts dropped significantly in subsequent applications. 

**Figure 10:**
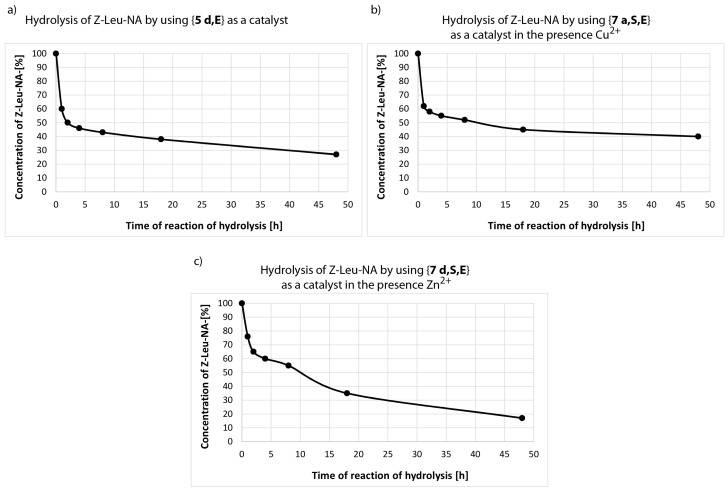
Progress of hydrolysis based on HPLC monitoring the concentration of Z-Leu-NA in the presence of N-hexadecanoyl-Glu-NH-C_6_H_4_-DMT-celulose {**5 d,E**} (**a**); in the presence of *N*-heptanoyl-Ser-Glu-NH-C_6_H_4_-DMT-celulose {**7 a,S,E**} as a catalyst in the presence Cu^2+^ (**b**); in the presence of *N*-heptanoyl-Ser-Glu-NH-C_6_H_4_-DMT-celulose {**7 d,S,E**} as a catalyst in the presence Zn^2+^ (**c**).

The additional studies on Z-Leu-NA hydrolysis were performed using synzymes selected as most active in reusability test: *N*-palmityl-Glu-NH-C_6_H_4_-DMT-celulose {**5 d,E**}, complex Cu^2+^ with *N*-heptanoyl-Ser-Glu-NH-C_6_H_4_-DMT-cellulose {**7 a,S,E**} and complex *N*-palmitoyl-Ser-Glu-NH- -C_6_H_4_-DMT-cellulose {**7 d,S,E**} with Zn^2+^ ion. The progress of reaction was monitored by HPLC method measuring the disappearance of the substrate peak (Z-Leu-NA, **10**) at Rt = 22.81 min accompanied by a steadily rising signal of Z-Leu-O-HNMM^+^(**11**) at Rt = 3.86 min and p-nitroaniline (**12**) at Rt = 8.72 min. The 16 mm × 3 mm strips (identical to those used in experiments on ELISA plates) were treated with 1.6 mL of the Z-Leu-NA solution at concentrations as used previously (with 10 times more substrate than was used in the screening test). Thus, 40 times more Z-Leu-NP was used in this test in relation to the amount of synzymes immobilized on cellulose. It was found that initial hydrolysis occurred very quickly and after 1 h 40% of the Z-Leu-NA was consumed in the case of reaction catalysed solely by {**5 d,E**} (without any metal ions) (Figure 10). However, later the process slowed and even after 48 h unreacted starting material still was observed. Experiments involving complex Cu^2+^ with {**7 a,S,E**} as the catalytic unit and 40 times excess of Z-Leu-NA, HPLC confirmed that the initial rate of reaction was very fast. After 1 h, substrate concentration fell to 60% (Figure 10b). However, later the reaction slowed and 40% of the substrate remained unchanged even after 48 h. The initial phase of Z-Leu-NA hydrolysis in the presence of **{7 d,S,E**} and Zn^2+^ ions was found to be relatively slow (after 1 h, as much as 80% of the starting material was unreacted) (Figure 10c ). However, despite a substantial fall in catalytic activity during the first 2 h period of transformation, after 48 h almost total substrate consumption was observed (the amount of unreacted Z-Leu-NA, based on HPLC, was less than 20%). 

The results for Z-Leu-NP consumption monitored by HPLC methods depicted in Figure 10a–c are in agreement with spectrophotometric determination of *p*-nitroaniline released after hydrolysis of substrate **10**. According to both approaches, very rapid hydrolysis during the early stage of transformation is accompanied by a substantial slowdown during more advanced stages. Monitoring substrate decay by the chromatographic method confirmed that hydrolysis continued to proceed, although its progress was slow. 

**Figure 11:**
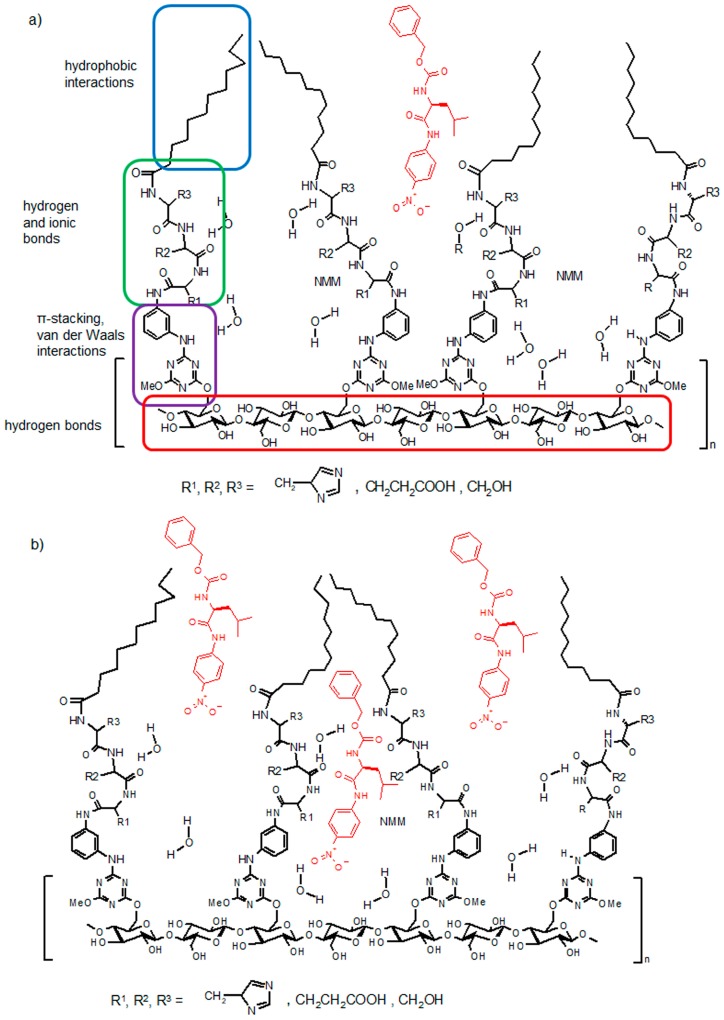

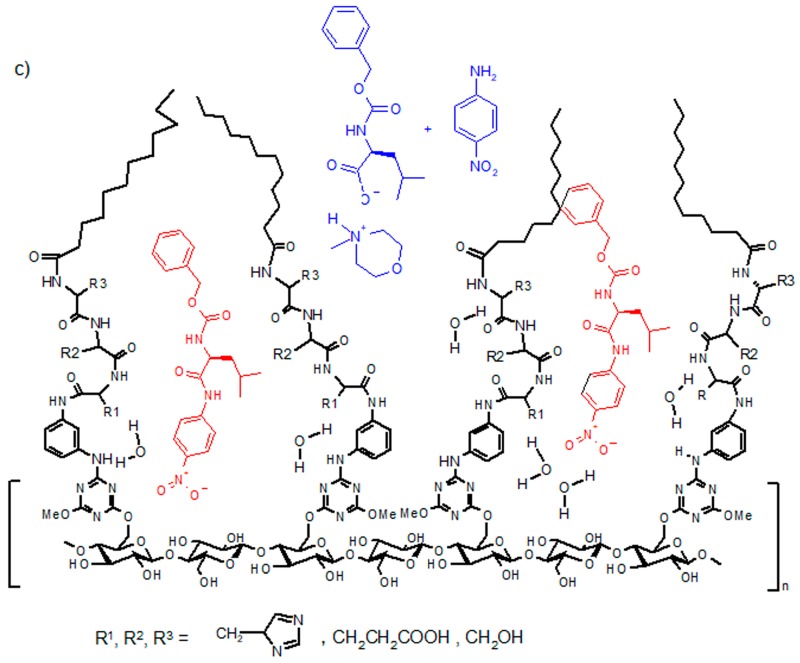
The proposed mechanism of action of synzymes able to split amide bond. (**a**) binding the substrate in the catalytic pocket; (**b**) closure of the substrate in the catalytic cavity and simultaneous formation of new binding sites; (**c**) release of reaction products and simultaneous closure of substrates in adjacent catalytic cavities.

The obtained results indicate that the mechanism of docking the ligand into the catalytic cavity, the catalytic cleavage process and the release of final products outside the catalytic cavity proceeds in a series of elementary processes. Each one can be implemented as a result of dynamic self-organization, which determines induced matching of the synzyme conformation to the requirements of the substrate and transition state. In the polar environment, the lipid chains of neighbouring podands immobilized on the cellulose strive to minimize contact with the polar and form a "lipid bundle" comprising at least two adjacent podands (Figure 11a). Thus, the "lipid bundles" are surrounded by a hydrophilic cavity with the exposed peptide fragment. The substrate having the ability to bind, is docking into the cavity, forces a change in the conformation of the N-lipidated peptides leading to the rearrangement of the conformation of the structure, the straighten out of "lipid bundles" and their re-formation, over the docked ligand. The nature of the interaction between the ligand and the peptide fragment implies that the most lipophilic part of the docking ligand will remain facing the lipidic fragment thereby facilitating the formation of binding interactions between the polar fragments of the ligand and the peptide. The exposure of the lipophilic part will favour the next re-formation of the "lipid bundle" which also includes a most hydrophobic fragment of the ligand structure. Such rearrangement will reveal another set of hydrophilic cavities ready for binding to the next ligand in the immediate vicinity of the cavity in which the ligand molecule has been already closed (Figure 11b).

Docking subsequent ligands forces is change the shape of the cavity. In direct contact with the functional groups of the amino acids of the catalytic triad remain the substrate of the reaction and the molecules of water and solvent. It should be expected that, with the favourable location of all reagents, the conditions for the chemical transformation of the substrate into the products will be met. The process of converting substrates into products in the microenvironment of the cavity may proceed at an increased rate due to the presence of the serine hydroxyl function, the carboxylic acid glutamic acid and/or finally the presence of the imidazole ring in the immediate vicinity of the amide group of the substrate. After hydrolysis and the formation of products, the next stage of the process involves their diffusion from the catalytically active site to the solution. This process may occur spontaneously and be additionally supported by the increasing entropy of the system after diffusion of products and by conformational changes of the podands forced by the docking of subsequent substrate molecules to immediately adjacent binding cavities (Figure 11c). The fact that synzymes can be repeatedly used confirms the proposed cycle of elementary physical and chemical processes, confirmed experimentally many times.

## 4. Conclusions

This study presents the preliminary results of a search for catalysts able to cleave amide bonds under biomimetic conditions, from among a library of 156 *N*-lipidated amino acids, dipeptides and tripeptides immobilized on cellulose. A highly diversified library of amide bond-cleaving catalysts was obtained. These chiral, biomimetic, biodegradable catalytic structures are affordable in both pseudo-enantiomeric forms. From a library of 156 synzymes assumed criterion max. absorbance above 0.8 was fulfilled by 8 serine rich *N*-lipidated tripeptides: {**9 b,S,S,S**}, {**9 d,S,S,S**}, {**9 d,H,S,S**}, {**9 b,S,E,S}**, {**9 d,S,E,S}**, {**9 a,E,H,S**}, {**9 d,H,S,E**} and {**9 c,S,S,H**}. The selected structures formed ***group A*** (catalytically active synzymes containing no additives in the catalytic pocket). Their activity can be easily modulated by modification of the peptide fragment, forming catalytic pocket and *N*-acylating lipid gating diffusion of the substrate into the binding cleft and dispersion of products. Their activity can be further enhanced by the presence of metal ions. It has been found that the presence of Cu^2+^ ions enhanced catalytic activity. Seven derivatives containing one amino acid residue in the catalytic centre were selected from the library of synzymes: {**5 b,S**}, {**5 c,S**}, {**5 d,S**}, {**5 b,E**}, {**5 c,E**}, {**5 d,E**}, {**5 d,H**}. Three derivatives containing dipeptide in the catalytic centre were chosen: {**7 a,E,E**}, {**7 a,S,E**}, {**7 c,E,H**}. Eight structures with tripeptide were selected: {**9 b,S,S,S**}, {**9 b,H,E,S**}, {**9 d,S,H,S**}, {**9 b,S,E,H**}, {**9 c,S,E,H**}, {**9 c,S,H,H**}, {**9 b,E,H,H**} and {**9 c,E,E,H**}. Selected *N*-lipidated amino acids, di- and tripeptides formed ***B group*** of synzymes containing Cu^2+^ ion in the catalytic pocket. Also the presence of Zn^2+^ ions enhanced catalytic activity. The most catalytically active synzymes, affording absorbance exceeding 0.8, included: four structures containing only one amino acid residue in the active centre {**5 c,S**}, {**5 b,E**}, {**5 d,H**} and {**5 c,H**}; six derivatives with a dipeptide fragment {**7 d,S,S**}, {**7 c,S,S**}, {**7 d,H,S**}, {**7 c,H,S**}, {**7 d,S,E**}, {**7 b,E,E**} and six derivatives containing tripeptide fragments in the active pocket {**9 d,S,H,S**}, {**9 a,E,H,S**}, {**9 d,H,S,H**}, {**9 d,S,E,H**}, **{9 b,S,H,H**}, {**9 a,E,S,H**}. The ***C group*** consisted of selected catalytically active synzymes containing Zn^2+^ ion in the catalytic pocket. 

It has been found that {**9 b,S,S,S**} is common to ***A*** and ***B groups*** and {**9 a,E,H,S**} is a member of ***A*** and ***C groups***. Common syzymes of ***B*** and ***C groups*** are: {**9 d,S,H,S**} and two structures with identical peptide fragments: SEH and SHH.

For the most active structures, rapid splitting of amide bonds was observed at the beginning of the hydrolytic process, followed by a slowdown of reaction rate at more advanced stages of transformation. This problem, most probably caused by precipitation of the product and clogging of the catalyst, may be resolved by modifying the reaction conditions. Analysis using HPLC showed that substrate consumption reached 60–83% even when the ratio of substrate **10** to catalyst was increased 10-fold (see Figure 10). The effect of the presence of a metal ion in the catalytic pocket was visible in the HPLC test. In the case of an identical catalytic pocket formed by dipeptide SE and various metal ions (Cu^2+^ and Zn^2+^), the substrate **10** consumption was 60 and 83%.

The further studies are ongoing to improve the catalytic efficiency by the modification of the structure of peptide fragment. The approach is also a promising method for constructing the other enzyme mimics using peptides immobilized on nanosized cellulose fibrils as the supramolecular framework. Considering their diversity and structural resemblance to the functional features of natural enzymes, the proposed supramolecules possibly represent a promising choice amongst enzyme mimics. Their other fascinating feature is biocompatibility of all basic components for example, cellulose, peptide and fatty acid used for construction of catalytic structures. This emerge their potential pharmaceutical utility as components in drug delivery systems, medical diagnostic, wound dressing materials and may others. Nevertheless, challenges that lie ahead to understand the relationship between the structural and functional aspects of proposed enzyme mimics is expected to be not trivial and obviously much work remains before these possibilities can be realized.

## Supplementary Materials

The following are available online at http://www.mdpi.com/1996-1944/12/4/578/s1, Figure S1: Measurement of Z-Leu-NA hydrolysis in a solution at pH = 8.5 using a library of synzymes: (a) sub-library of synzymes with serine residue at C-terminal positions, (b) sub-library of synzymes with glutamic acid residue at C-terminal positions, (c) sub-library of synzymes with histidine residue at C-terminal positions, Figure S2: Measurement of Z-Leu-NA hydrolysis using the 9 most effective synzymes at pH 8.5 (third catalytic cycle), Figure S3: Measurement of Z-Leu-NA hydrolysis in a solution at pH = 8.5 using a library of synzymes with Cu2+ ions docked in the catalytic pocket: (a) sub-library of synzymes with serine residue at C-terminal positions, (b) sub-library of synzymes with glutamic acid residue at C-terminal positions, (c) sub-library of synzymes with histidine residue at C-terminal positions, Figure S4: Measurement of Z-Leu-NA hydrolysis at pH 8.5 using the most effective synzymes with Cu2+ docked in the active pocket (third catalytic cycle), Figure S5: Measurement of Z-Leu-NA hydrolysis in a solution at pH = 8.5 using a library of synzymes with Zn2+ ions docked in the catalytic pocket: (a) sub-library with serine residue at C-terminal positions, (b) sub-library with glutamic acid residue at C-terminal positions, (c) sub-library with histidine residue at C-terminal positions, Figure S6: Measurement of Z-Leu-NA hydrolysis at pH 8.5 using the most effective synzymes with Zn2+ ions docked in the active pocket (third catalytic cycle).
