# Supplementary material for: N-Lipidated Amino Acids and Peptides Immobilized on Cellulose Able to Split Amide Bonds
Source: Materials (Basel). 2019 Feb 14;12(4):578. doi: 10.3390/ma12040578 (PMC6416662; doi:10.3390/ma12040578)
Supplement: Supplementary file 1 [file materials-12-00578-s001.pdf]

materials

MDPI

Article

N-Lipidated Amino Acids and Peptides Immobilized on Cellulose Able to Split Amide Bonds

Justyna Fraczyk * and Zbigniew J. Kaminski

Institute of Organic Chemistry, Lodz University of Technology, Zeromskiego 116, 90-924 Lodz, Poland; justyna.fraczyk@p.lodz.pl, zbigniew.kaminski@p.lodz.pl

* Correspondence: justyna.fraczyk@p.lodz.pl; Tel.: +48-426-313-321

Received: 26 January 2019; Accepted: 11 February 2019; Published: date

a)

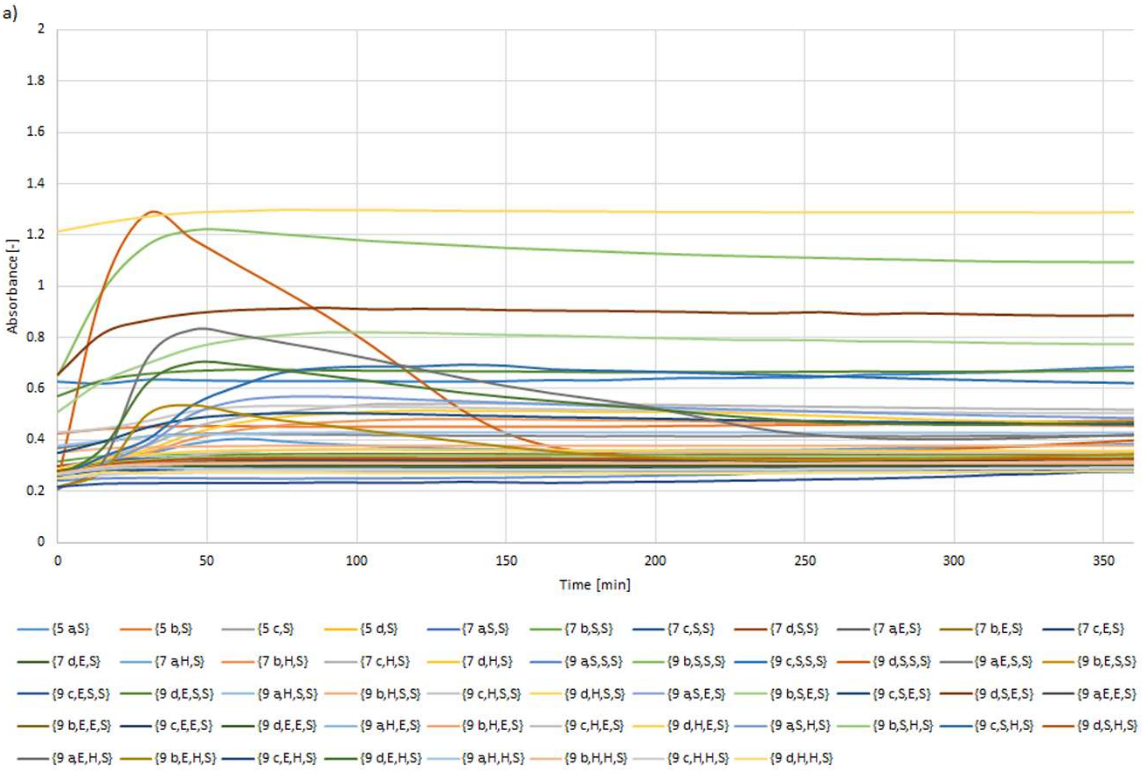

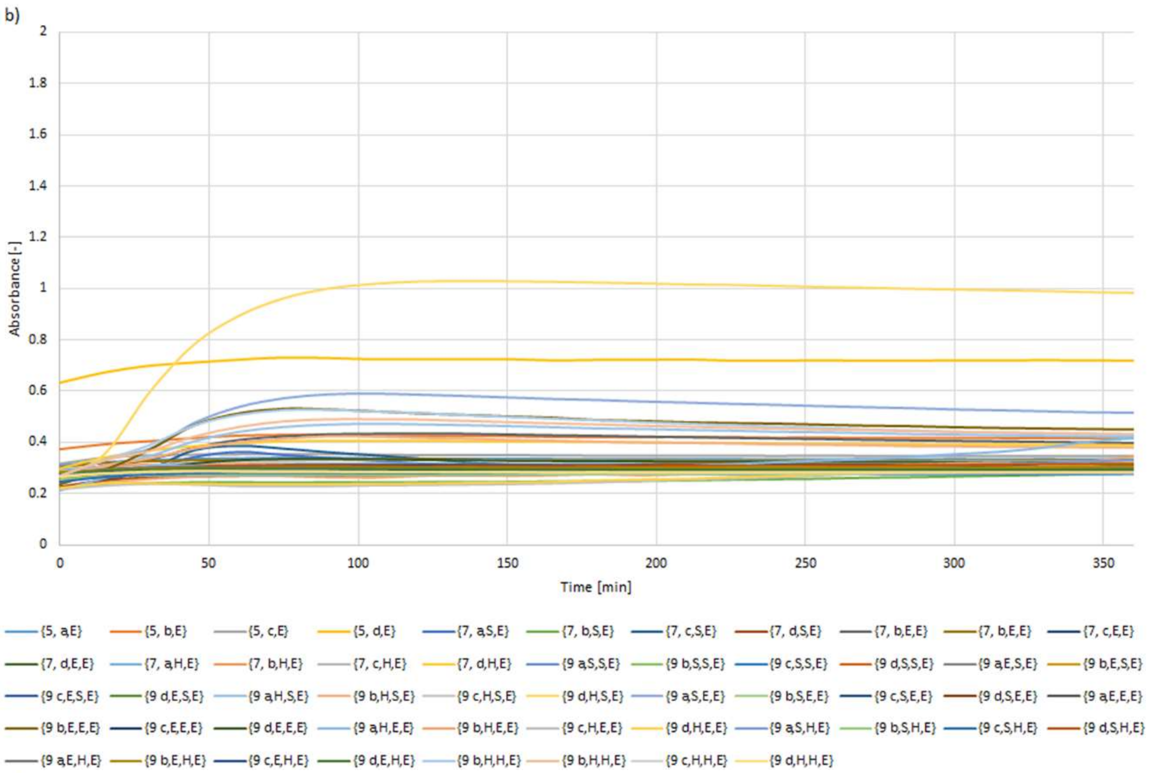

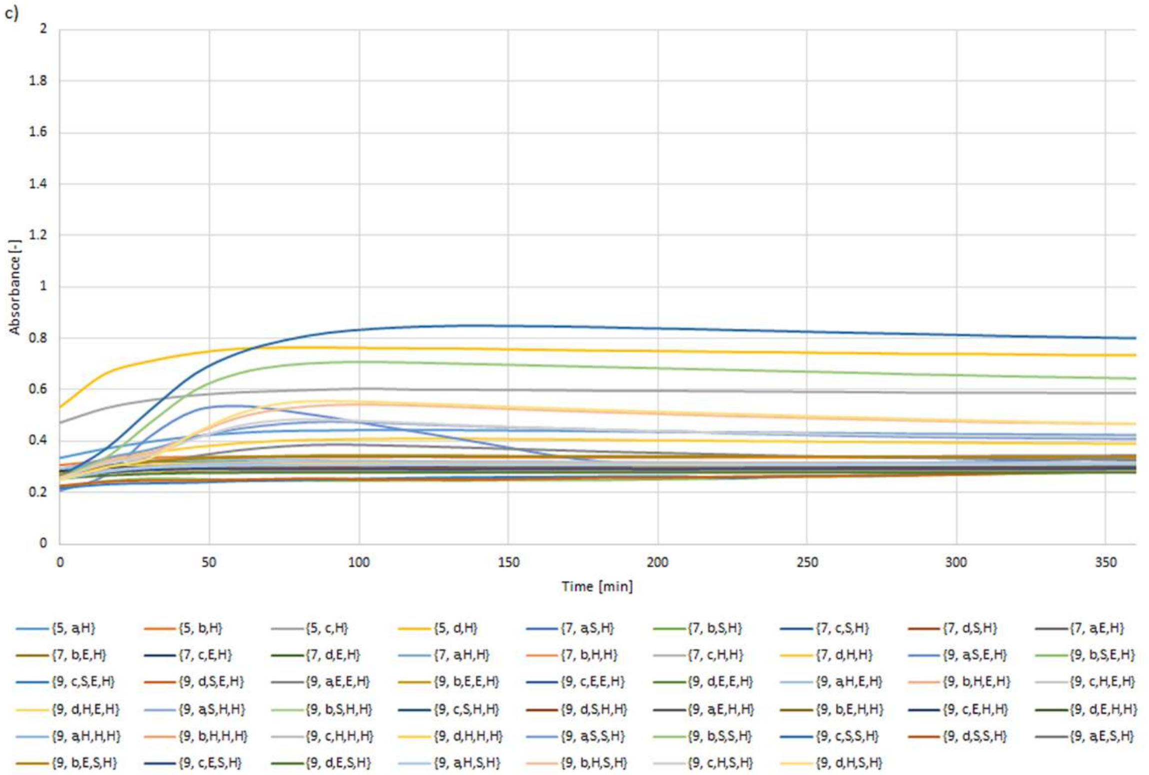

Figure S1. Measurement of Z-Leu-NA hydrolysis in a solution at pH = 8.5 using a library of synzymes: (**a**) sub-library of synzymes with serine residue at *C*-terminal positions, (**b**) sub-library of synzymes with glutamic acid residue at *C*-terminal positions, (**c**) sub-library of synzymes with histidine residue at *C*-terminal positions.

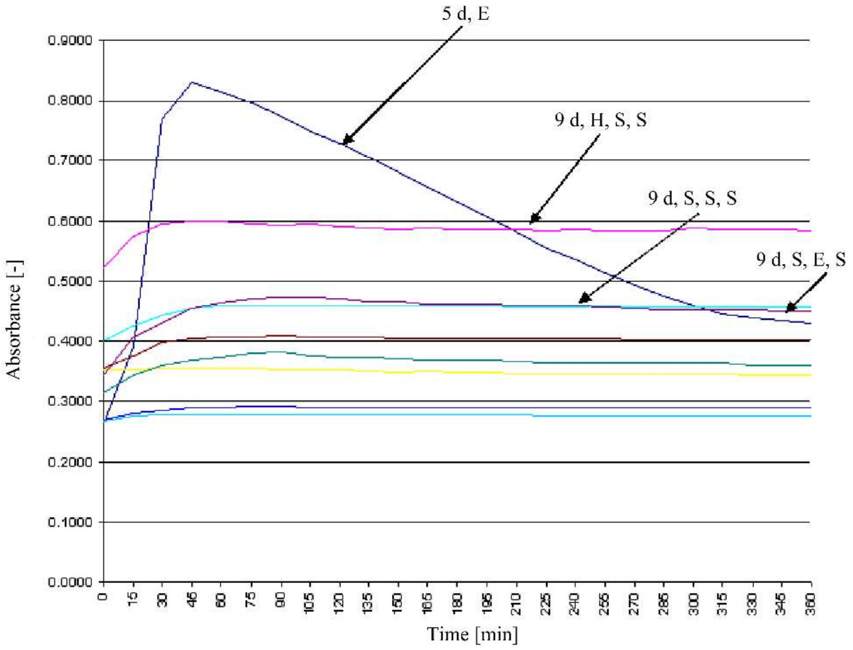

Figure S2. Measurement of Z-Leu-NA hydrolysis using the 9 most effective synzymes at pH 8.5 (third catalytic cycle).

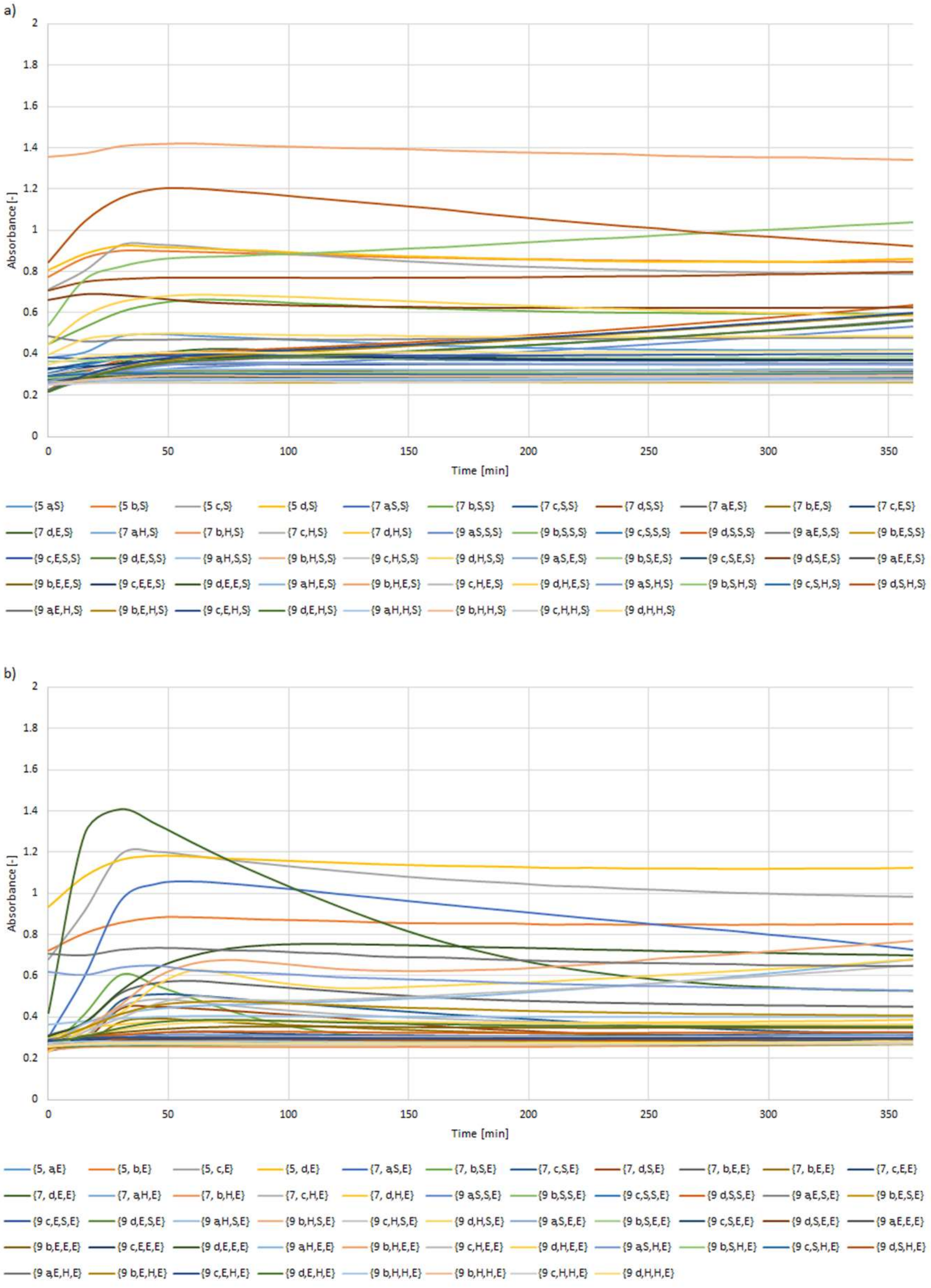
a)
Absorbance [-]
Time [min]
{5 a,S}
{5 b,S}
{5 c,S}
{5 d,S}
{7 a,S,S}
{7 b,S,S}
{7 c,S,S}
{7 d,S,S}
{7 a,E,S}
{7 b,E,S}
{7 c,E,S}
{7 d,E,S}
{7 a,H,S}
{7 b,H,S}
{7 c,H,S}
{7 d,H,S}
{9 a,S,S,S}
{9 b,S,S,S}
{9 c,S,S,S}
{9 d,S,S,S}
{9 a,E,S,S}
{9 b,E,S,S}
{9 c,E,S,S}
{9 d,E,S,S}
{9 a,H,S,S}
{9 b,H,S,S}
{9 c,H,S,S}
{9 d,H,S,S}
{9 a,S,E,S}
{9 b,S,E,S}
{9 c,S,E,S}
{9 d,S,E,S}
{9 a,E,E,S}
{9 b,E,E,S}
{9 c,E,E,S}
{9 d,E,E,S}
{9 a,H,E,S}
{9 b,H,E,S}
{9 c,H,E,S}
{9 d,H,E,S}
{9 a,S,H,S}
{9 b,S,H,S}
{9 c,S,H,S}
{9 d,S,H,S}
{9 a,E,H,S}
{9 b,E,H,S}
{9 c,E,H,S}
{9 d,E,H,S}
{9 a,H,H,S}
{9 b,H,H,S}
{9 c,H,H,S}
{9 d,H,H,S}
b)
Absorbance [-]
Time [min]
{5, a,E}
{5, b,E}
{5, c,E}
{5, d,E}
{7, a,S,E}
{7, b,S,E}
{7, c,S,E}
{7, d,S,E}
{7, b,E,E}
{7, b,E,E}
{7, c,E,E}
{7, d,E,E}
{7, a,H,E}
{7, b,H,E}
{7, c,H,E}
{7, d,H,E}
{9 a,S,S,E}
{9 b,S,S,E}
{9 c,S,S,E}
{9 d,S,S,E}
{9 a,E,S,E}
{9 b,E,S,E}
{9 c,E,S,E}
{9 d,E,S,E}
{9 a,H,S,E}
{9 b,H,S,E}
{9 c,H,S,E}
{9 d,H,S,E}
{9 a,S,E,E}
{9 b,S,E,E}
{9 c,S,E,E}
{9 d,S,E,E}
{9 a,E,E,E}
{9 b,E,E,E}
{9 c,E,E,E}
{9 d,E,E,E}
{9 a,H,E,E}
{9 b,H,E,E}
{9 c,H,E,E}
{9 d,H,E,E}
{9 a,S,H,E}
{9 b,S,H,E}
{9 c,S,H,E}
{9 d,S,H,E}
{9 a,E,H,E}
{9 b,E,H,E}
{9 c,E,H,E}
{9 d,E,H,E}
{9 b,H,H,E}
{9 b,H,H,E}
{9 c,H,H,E}
{9 d,H,H,E}

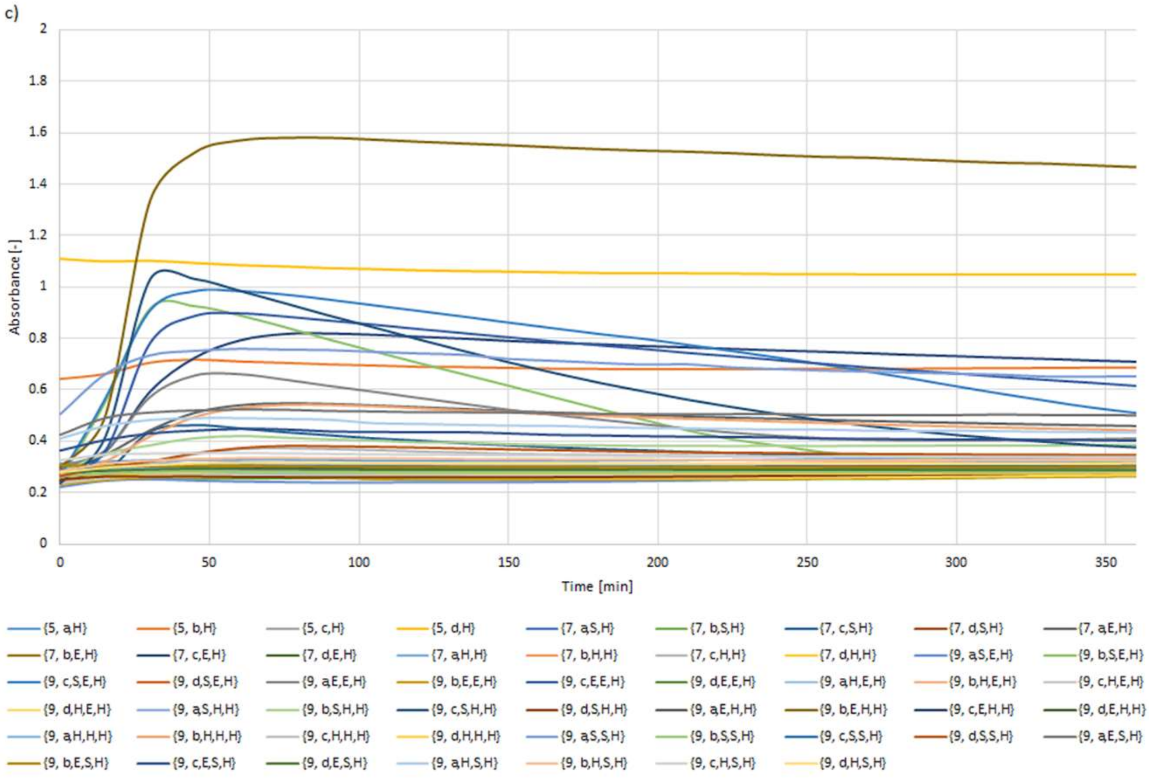

Figure S3. Measurement of Z-Leu-NA hydrolysis in a solution at pH = 8.5 using a library of synzymes with Cu^{2+} ions docked in the catalytic pocket: (**a**) sub-library of synzymes with serine residue at *C*-terminal positions, (**b**) sub-library of synzymes with glutamic acid residue at *C*-terminal positions, (**c**) sub-library of synzymes with histidine residue at *C*-terminal positions.

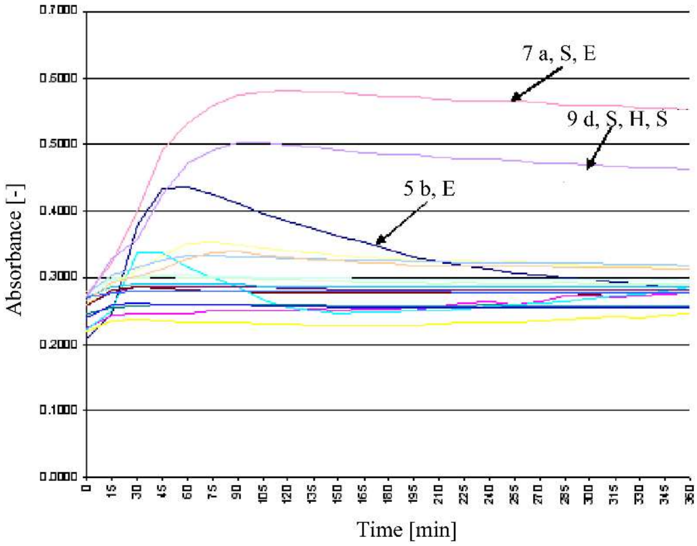

Figure S4. Measurement of Z-Leu-NA hydrolysis at pH 8.5 using the most effective synzymes with Cu^{2+} docked in the active pocket (third catalytic cycle).

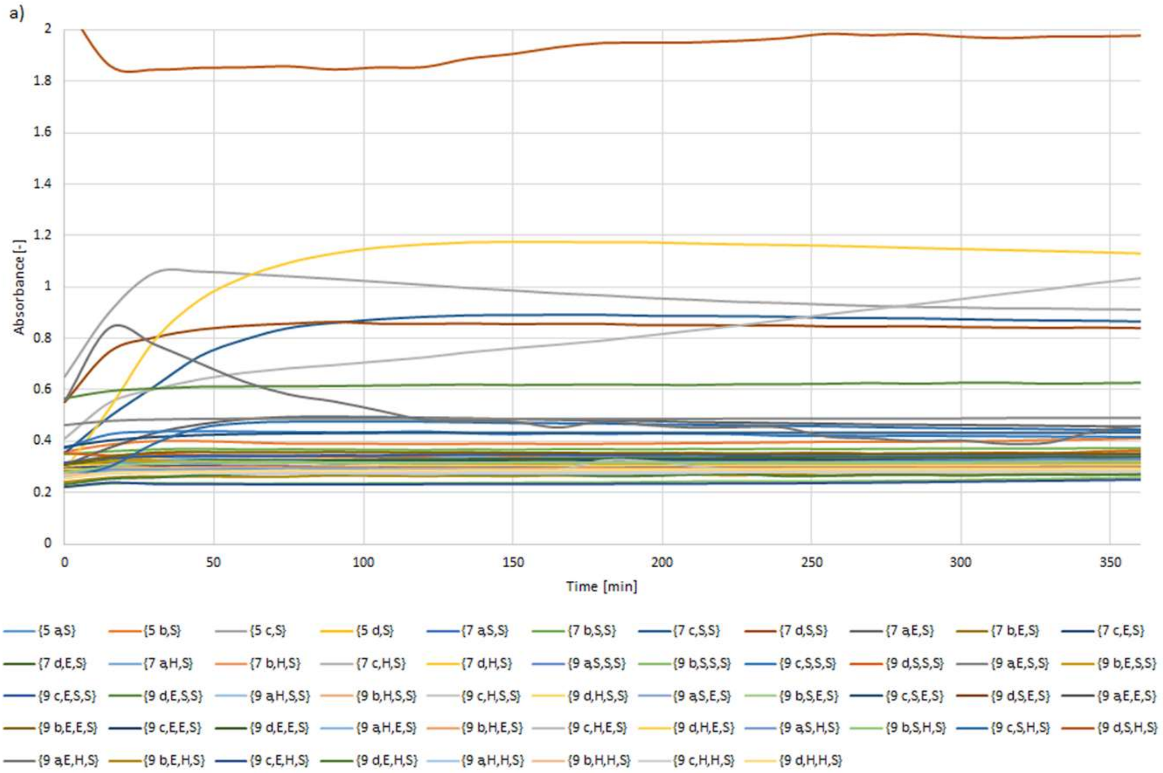
a)
Absorbance [-]
0
0.2
0.4
0.6
0.8
1
1.2
1.4
1.6
1.8
2
0
50
100
150
200
250
300
350
Time [min]
{5 a,S}
{5 b,S}
{5 c,S}
{5 d,S}
{7 a,S,S}
{7 b,S,S}
{7 c,S,S}
{7 d,S,S}
{7 a,E,S}
{7 b,E,S}
{7 c,E,S}
{7 d,E,S}
{7 a,H,S}
{7 b,H,S}
{7 c,H,S}
{7 d,H,S}
{9 a,S,S,S}
{9 b,S,S,S}
{9 c,S,S,S}
{9 d,S,S,S}
{9 a,E,S,S}
{9 b,E,S,S}
{9 c,E,S,S}
{9 d,E,S,S}
{9 a,H,S,S}
{9 b,H,S,S}
{9 c,H,S,S}
{9 d,H,S,S}
{9 a,S,E,S}
{9 b,S,E,S}
{9 c,S,E,S}
{9 d,S,E,S}
{9 a,E,E,S}
{9 b,E,E,S}
{9 c,E,E,S}
{9 d,E,E,S}
{9 a,H,E,S}
{9 b,H,E,S}
{9 c,H,E,S}
{9 d,H,E,S}
{9 a,S,H,S}
{9 b,S,H,S}
{9 c,S,H,S}
{9 d,S,H,S}
{9 a,E,H,S}
{9 b,E,H,S}
{9 c,E,H,S}
{9 d,E,H,S}
{9 a,H,H,S}
{9 b,H,H,S}
{9 c,H,H,S}
{9 d,H,H,S}

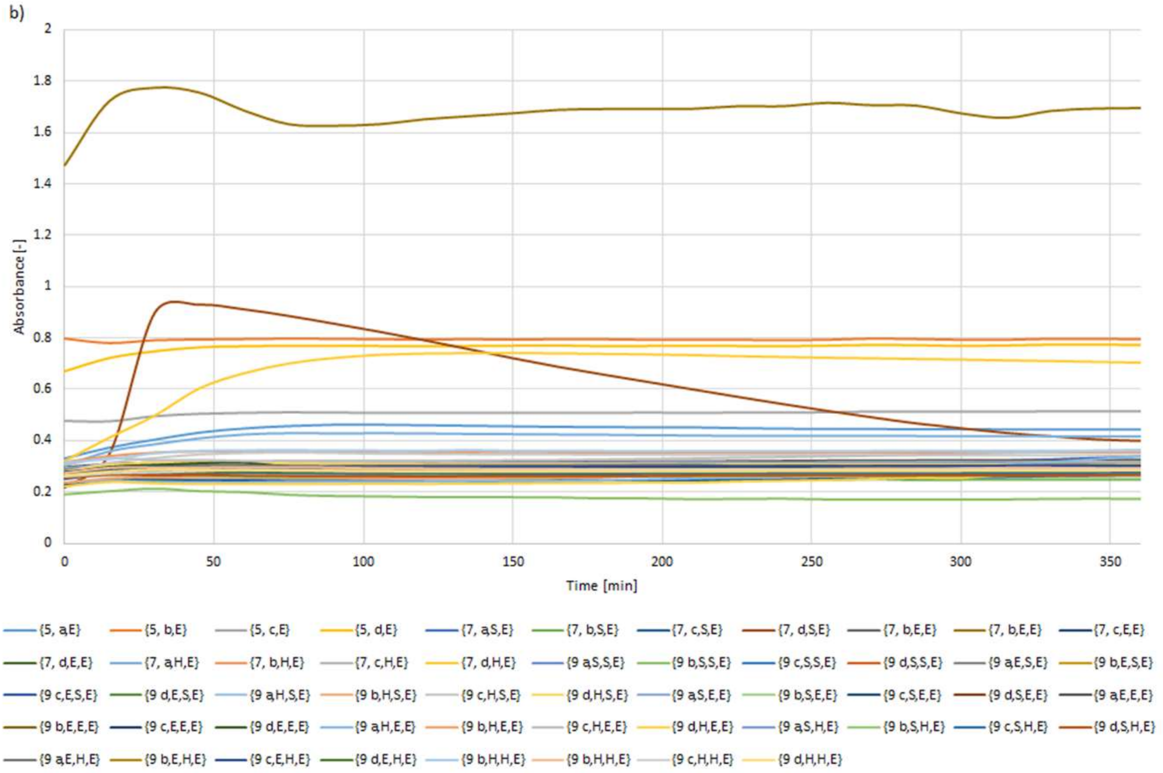
b)
Absorbance [-]
0
0.2
0.4
0.6
0.8
1
1.2
1.4
1.6
1.8
2
0
50
100
150
200
250
300
350
Time [min]
{5, a,E}
{5, b,E}
{5, c,E}
{5, d,E}
{7, a,S,E}
{7, b,S,E}
{7, c,S,E}
{7, d,S,E}
{7, b,E,E}
{7, b,E,E}
{7, c,E,E}
{7, d,E,E}
{7, a,H,E}
{7, b,H,E}
{7, c,H,E}
{7, d,H,E}
{9 a,S,S,E}
{9 b,S,S,E}
{9 c,S,S,E}
{9 d,S,S,E}
{9 a,E,S,E}
{9 b,E,S,E}
{9 c,E,S,E}
{9 d,E,S,E}
{9 a,H,S,E}
{9 b,H,S,E}
{9 c,H,S,E}
{9 d,H,S,E}
{9 a,S,E,E}
{9 b,S,E,E}
{9 c,S,E,E}
{9 d,S,E,E}
{9 a,E,E,E}
{9 b,E,E,E}
{9 c,E,E,E}
{9 d,E,E,E}
{9 a,H,E,E}
{9 b,H,E,E}
{9 c,H,E,E}
{9 d,H,E,E}
{9 a,S,H,E}
{9 b,S,H,E}
{9 c,S,H,E}
{9 d,S,H,E}
{9 a,E,H,E}
{9 b,E,H,E}
{9 c,E,H,E}
{9 d,E,H,E}
{9 b,H,H,E}
{9 b,H,H,E}
{9 c,H,H,E}
{9 d,H,H,E}

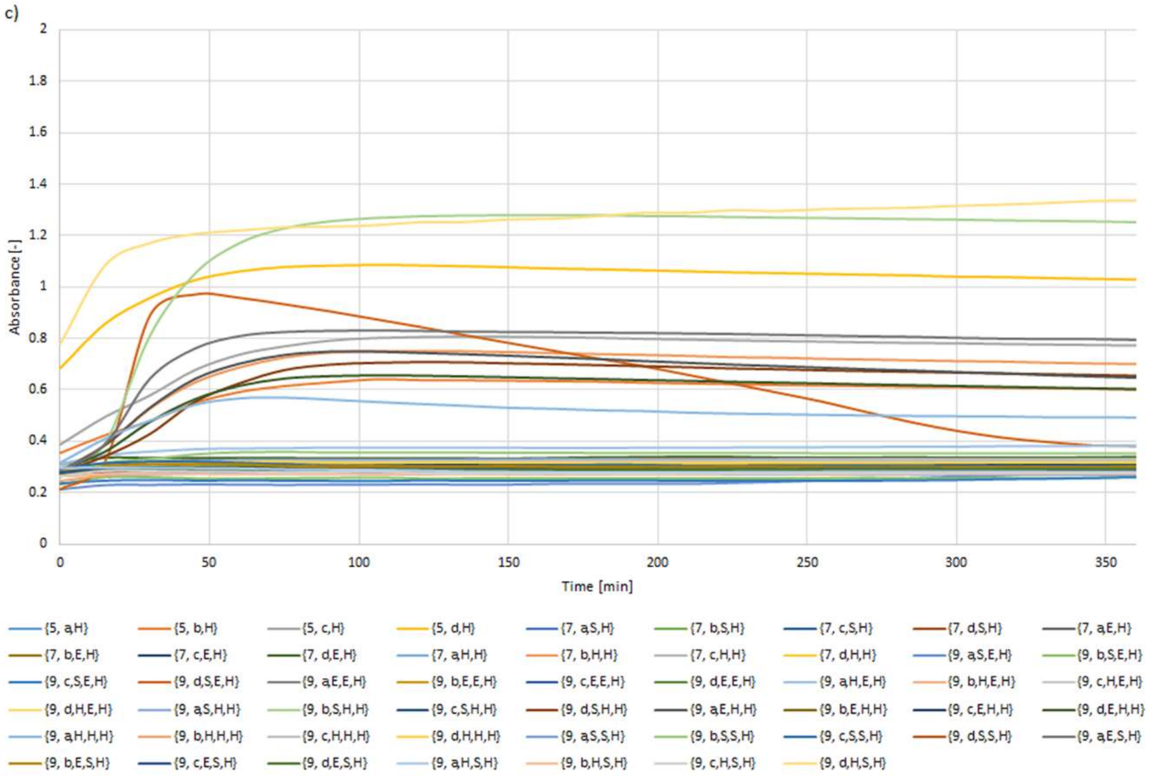

Figure S5. Measurement of Z-Leu-NA hydrolysis in a solution at pH = 8.5 using a library of synzymes with Zn^{2+} ions docked in the catalytic pocket: (**a**) sub-library with serine residue at *C*-terminal positions, (**b**) sub-library with glutamic acid residue at *C*-terminal positions, (**c**) sub-library with histidine residue at *C*-terminal positions.

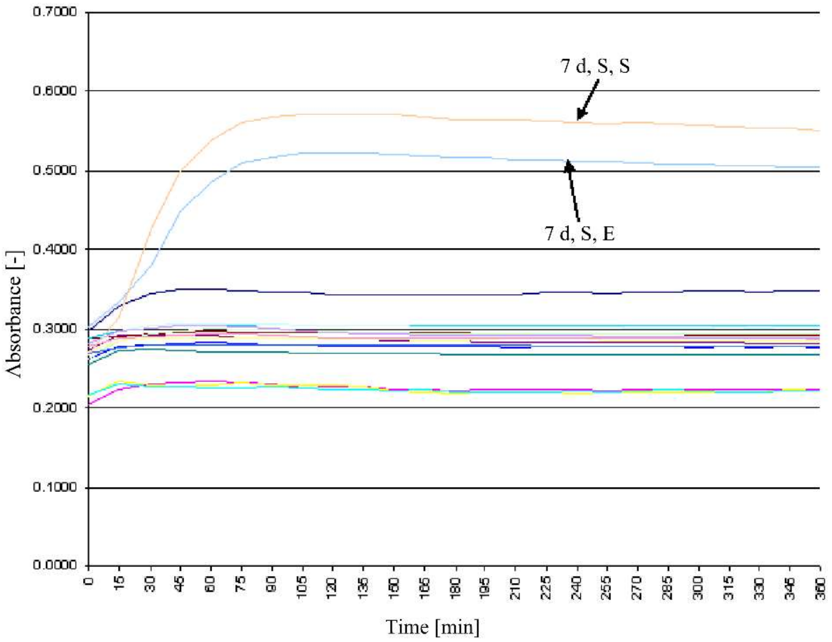

Figure S6. Measurement of Z-Leu-NA hydrolysis at pH 8.5 using the most effective synzymes with Zn^{2+} ions docked in the active pocket (third catalytic cycle).
